# BSCL2/Seipin deficiency in hearts causes cardiac energy deficit and dysfunction via inducing excessive lipid catabolism

**DOI:** 10.1002/ctm2.736

**Published:** 2022-04-05

**Authors:** Hongyi Zhou, Jie Li, Huabo Su, Ji Li, Todd A. Lydic, Martin E Young, Weiqin Chen

**Affiliations:** ^1^ Department of Physiology Medical College of Georgia at Augusta University Augusta Georgia USA; ^2^ Vascular Biology Center Medical College of Georgia at Augusta University Augusta Georgia USA; ^3^ Department of Surgery Morsani College of Medicine University of South Florida Tampa Florida USA; ^4^ Department of Physiology Michigan State University East Lansing Michigan USA; ^5^ Department of Medicine University of Alabama at Birmingham Birmingham Alabama USA

**Keywords:** BSCL2/Seipin, heart failure, lipid metabolism, lipidomics

## Abstract

**Background:**

Heart failure (HF) is one of the leading causes of death worldwide and is associated with cardiac metabolic perturbations. Human Type 2 Berardinelli‐Seip Congenital Lipodystrophy (BSCL2) disease is caused by mutations in the *BSCL2* gene. Global lipodystrophic *Bscl2^−/−^* mice exhibit hypertrophic cardiomyopathy with reduced cardiac steatosis. Whether BSCL2 plays a direct role in regulating cardiac substrate metabolism and/or contractile function remains unknown.

**Methods:**

We generated mice with cardiomyocyte‐specific deletion of *Bscl2* (*Bscl2^cKO^*) and studied their cardiac substrate utilisation, bioenergetics, lipidomics and contractile function under baseline or after either a treatment regimen using fatty acid oxidation (FAO) inhibitor trimetazidine (TMZ) or a prevention regimen with high‐fat diet (HFD) feeding. Mice with partial ATGL deletion and cardiac‐specific deletion of *Bscl2* were also generated followed by cardiac phenotyping.

**Results:**

Different from hypertrophic cardiomyopathy in *Bscl2^−/−^* mice, mice with cardiac‐specific deletion of *Bscl2* developed systolic dysfunction with dilation. Myocardial BSCL2 deletion led to elevated ATGL expression and FAO along with reduced cardiac lipid contents. Cardiac dysfunction in *Bscl2^cKO^* mice was independent of mitochondrial dysfunction and oxidative stress, but associated with decreased metabolic reserve and ATP levels. Importantly, cardiac dysfunction in *Bscl2^cKO^* mice could be partially reversed by FAO inhibitor TMZ, or prevented by genetic abolishment of one ATGL allele or HFD feeding. Lipidomic analysis further identified markedly reduced glycerolipids, glycerophospholipids, NEFA and acylcarnitines in *Bscl2^cKO^* hearts, which were partially normalised by TMZ or HFD.

**Conclusions:**

We identified a new form of cardiac dysfunction with excessive lipid utilisation which ultimately causes cardiac substrate depletion and bioenergetics failure. Our findings also uncover a crucial role of BSCL2 in controlling cardiac lipid catabolism and contractile function and provide novel insights into metabolically treating energy‐starved HF using FAO inhibitor or HFD.

## Graphical abstract

Cardiac deletion of BSCL2 causes ATGL overexpression, excessive fatty acid oxidation (FAO), drastic reduction of cardiac lipidome and ultimately energetic and contractile dysfunction in mice. Inhibiting FAO by partial ATGL deletion or trimetazidine and increasing lipid supply via high fat diet (HFD) feeding alleviate cardiac dysfunction caused by BSCL2 deficiency. BSCL2 is a novel protein indispensable for cardiac lipid metabolism and function.

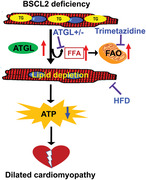

## 1 INTRODUCTION

Heart failure (HF) is one of the leading causes of morbidity and mortality worldwide. In healthy individuals, the heart exhibits striking metabolic flexibility, being capable of utilising carbohydrate, lipid, amino acids and/or ketone bodies to meet energetic demands, cellular constituent turnover and metabolic signalling; these processes are critical for maintenance of mechanical work. Oxidation of fatty acids (FAs) predominates, accounting for 60–70% of myocardial oxygen consumption.^1^ In patients with hypertrophied and failing hearts, derangements of substrate utilisation include an increased reliance on glycolysis concomitant with an overall reduced oxidative metabolism.^2^ The severely failing heart usually demonstrates a lower concentration of ATP, supporting the concept that energy starvation contributes significantly to the pathogenesis of HF.^3^ Indeed, there is a striking correlation between cardiac energetic status and survival in HF patients.^4^ Thus, targeting metabolic processes in the heart may represent a promising way to develop new therapeutic approaches for HF.

Cardiac lipid metabolism is precisely controlled to maintain a balance between FA uptake, triglyceride (TG) synthesis, TG hydrolysis and FA oxidation (FAO).^5^ Imbalances in these processes are commonly seen in obese and diabetic patients and animal models, which are associated with cardiac lipotoxicity (i.e., accumulation of toxic lipid intermediates) and contractile dysfunction.^5^ Recent preclinical and clinical evidence argues for an important role of adipose triglyceride lipase (ATGL)‐mediated cardiac lipolysis in promoting mitochondrial FAO and ATP production thus contractile function.^6^ Constitutive *Atgl* ***^−/−^*** mice develop severe cardiac steatosis and HF, associated with a high mortality.^7^ Conversely, cardiomyocyte‐specific overexpression of ATGL maintains normal cardiac function in lean mice and reduces cardiac TG content and improves cardiac function during diabetes and obesity.^8, 9^ The precise mechanisms linking cardiac lipid catabolism with contractile dysfunction remain to be further explored.

Type 2 Berardinelli‐Seip Congenital Lipodystrophy (BSCL2) is caused by mutations in a gene called *BSCL2* (also called Seipin), a highly conserved endoplasmic reticulum (ER) protein expressed in most tissues, with the highest levels in testis, neuronal and adipose tissue.^10^, ^11^ In addition to metabolic abnormalities including insulin resistance and type 2 diabetes,^12^, ^13^ patients with BSCL2 develop progressive hypertrophic cardiomyopathy, highlighted by concentric left ventricular hypertrophy and cardiac failure.^14^, ^15^, ^16^, ^17^ Global *Bscl2*‐deficient (*Bscl2* **^−/−^**) mice recapitulate human BSCL2 disease, exhibiting congenital lipodystrophy and severe insulin resistance.^18^, ^19^, ^20^ Various molecular functions of BSCL2 have been proposed, ranging from regulating lipid droplet (LD) biogenesis^21^, ^22^ to mitochondrial metabolism.^23^, ^24^ BSCL2 forms oligomers of 10–12 subunits and plays crucial roles in lipid transfer and/or LD formation.^25^, ^26^ BSCL2 has been shown to interact with 1‐acylglycerol‐3‐phosphate O‐acyltransferase 2 (AGPAT2),^27^ LIPIN1,^28^ glycerol‐3‐phosphate acyltransferase 3 (GPAT3),^29^ and Promethin.^30^, ^31^ We and others demonstrate that BSCL2 plays a key role in regulating cyclic AMP/protein kinase A (cAMP/PKA)‐mediated TG lipolysis essential for white adipocyte differentiation.^18^, ^20^ Deletion of BSCL2 in mature white and brown adipose tissue also triggers cAMP/PKA‐mediated lipolysis and FAO resulting in adipose tissue loss.^32^, ^33^, ^34^ Recently, we reported that *Bscl2* **^−/−^** mice develop hypertrophic cardiomyopathy with reduced cardiac steatosis.^35^ Despite the relatively low expression of BSCL2 in murine hearts, whether BSCL2 plays a cell‐autonomous role in modulating cardiac lipid metabolism and function has not been fully addressed.

The present study highlights a new form of metabolic cardiomyopathy caused by deletion of BSCL2 in hearts. Cardiomyocyte‐specific deletion of BSCL2 enhanced ATGL expression and FAO, resulting in markedly reduced cardiac lipid reserve, associated with compromised ATP production and contractile dysfunction. Inhibition of FAO or supplying FAs by high‐fat diet (HFD) feeding partially alleviated cardiac energetic stress and augmented contractile function. These findings improve our understanding of how perturbations in lipid utilisation/storage contribute towards HF development and provide preclinical insights into metabolic treatment of HF.

## 2 MATERIALS AND METHODS

### 2.1 Mice

*Cre+;Bscl2^f/f^* mice (i.e., *Bscl2^cKO^*) were generated by breeding *Bscl2^f/f^* mice^18^ with transgenic mice expressing Cre recombinase under the cardiac‐specific alpha myosin‐heavy chain 6 (*Myh6*) promoter (JAX Cat#:011038).^36^ All mice were under C57BL/6J background. The *Myh6‐Cre* mice (i.e., *Cre+;Bscl2^w/w^*) were included as controls when assessing cardiac and mitochondrial functions. *Cre−;Bscl2^f/f^* mice were used as controls (Ctrl) for the majority of the studies. *Atgl^+/–^;Bscl2^cKO^* mice were generated by breeding heterozygous *Atgl^+/–^* mice (B6;129P2‐Pnpla2^tm1Rze^/J, Jackson stock #: 019003) with *Bscl2^cKO^* mice. Littermates generated from *Atgl^+/–^;Bscl2^f/f^* and *Atgl^+/–^;Bscl2^cKO^* mating [i.e., *Atgl^+/+^;Bscl2^f/f^*;*Myh6‐Cre*− (Ctrl), *Atgl^+/+^;Bscl2^f/f^;Myh6‐Cre+* (*B^cKO^*) and *Atgl^+/–^;Bscl2^f/f^;Myh6‐Cre+* (*A^h^B^cKO^*)] were used for all experiments. Most experiments were performed in ad libitum male mice with at least two independent cohorts. Some experiments were also repeated in female mice. To inhibit mitochondrial β‐oxidation, 6‐month‐old Ctrl and *Bscl2^cKO^* mice were i.p. injected with PBS or trimetazidine (TMZ) at 15 mg/kg/day for 6 weeks, a dose that does not induce whole‐body insulin resistance.^37^ Ctrl and *Bscl2^cKO^* mice were fed a 60% HFD (Research Diets; D12492) starting at 3 months of age for a duration of 3 months. Most experiments were performed in ad libitum male mice and repeated in female mice. Body compositions were measured using a Bruker small animal NMR system (Bruker minispec LF90II). Transthoracic 2D and M‐mode echocardiography was performed with a VisualSonics Vevo 2100 equipped with a 30 MHz probe (VisualSonics). Mice were initially anaesthetised with a 3% isoflurane, followed by maintenance at 1–2%. Cardiac gravimetric and histological measurements were performed as described elsewhere.^35^ All mice were housed in the central animal facility with room temperature controlled at 21°C, and an artificial 12 h:12 h light:dark cycle (lights on at 06:00 am). Mice were directly sacrificed by cervical dislocation and hearts were rapidly excised. In ex vivo perfused working heart experiments, hearts were rapidly excised following anaesthesia via intraperitoneal ketamine/xylazine (80/10 mg/kg) injection. 

### 2.2 Myocardial substrate utilisation and contractile function in isolated working mouse hearts

All hearts from 3‐month‐old male Ctrl and *Bscl2^cKO^* mice were prepared and perfused in the working mode (non‐recirculating manner) for 30 min with a preload of 12.5 mmHg and an afterload of 50 mmHg as described.^38^ Standard Krebs–Henseleit buffer was supplemented with 8 mM glucose, 0.4 mM oleate conjugated to 3% BSA (fraction V, FA‐free; dialysed), 10 μU/ml insulin (basal/fasting concentration), 0.05 mM l‐carnitine and 0.13 mM glycerol. Metabolic fluxes were assessed through the use of distinct radiolabelled tracers: (1) [U‐^14^C]‐glucose (0.12 mCi/L from MP Biomedicals; glucose oxidation) and (2) [9, 10–^3^H]‐oleate (0.067 mCi/L from Sigma–Aldrich; β‐oxidation). Measures of cardiac metabolism (e.g., oleate and glucose oxidation and oxygen consumption) and function (e.g., cardiac power) were determined. At the end of the perfusion period, hearts were snap‐frozen and stored at −80°C prior to analysis. Data were presented as steady‐state values (i.e., the mean of the last two time points during a distinct perfusion condition for each individual heart).

### 2.3 Lipidomic analysis by high‐resolution/accurate mass spectrometry and tandem mass spectrometry

Total lipids from frozen ventricles were extracted and resuspended in isopropanol:methanol:chloroform (4:2:1, v:v:v) containing 20 mM ammonium formate followed by untargeted lipidomic analysis. Relative quantification of abundances between samples was performed by normalising target lipid ion peak areas to the PC (14:0/14:0) internal standard followed by normalisation to tissue weights as previously described.^35^

### 2.4 FA and glucose oxidation assays

FAO and glucose oxidation reaction assays with LV homogenates were prepared and carried out as detailed previously.^39^

### 2.5 Mitochondrial isolation and measurement of mitochondrial respiration

Fresh ventricles were isolated and minced for mitochondrial isolation and measurement of mitochondrial respiration by XFe24 Extracellular Flux Analyzer (Seahorse Bioscience) as previously described.^34^ See Supplemental Materials and Methods for details.

### 2.6 Tissue ATP measurement

ATP content was determined by using ATP Bioluminescent Assay Kit (FL‐AA; Sigma–Aldrich, Saint Louis, MO, USA) according to the manufacturer's procedure. Briefly, frozen heart tissues were homogenised in cold 10% trichloroacetic acid buffer, centrifuged at 5000×*g* for 10 min at 4°C followed by neutralisation with 50 mM Tris–acetate (pH 7.8). The ATP content was then determined by a microplate reader with luminescence luminometer (FLUOstar Omega; BMG Labtech). Data were normalised to tissue weight.

### 2.7 RNA isolation and quantitative real‐time PCR

Total RNA was extracted with Trizol Reagent (Thermo Fisher) and reverse‐transcribed using MLV‐V reverse transcriptase using random primers (Invitrogen). Real‐time quantitative RT‐PCR was performed on the Strategene MX3005 system. Data were normalised to two housekeeping genes (*Actb* and *36B4*) based on Genorm algorithm (medgen.ugent.be/genorm/) and expressed as fold changes. All tissue gene expression studies were performed in non‐fasted mice. qRT‐PCR primers were listed in Table S1.

### 2.8 Immunoblotting

Protein extraction, determination of protein concentrations, Western blotting and quantification were performed as previously described.^35^ Specific antibodies were listed in Supplemental Materials.

### 2.9 Statistical analysis

Quantitative data were presented as means ± SEM. Statistical comparisons were made by using unpaired *t‐*test, one‐way ANOVA or two‐way ANOVA followed by Tukey's post‐hoc tests or multiple *t‐*tests after correction using the Holm–Sidak method using the built‐in statistics of GraphPad Prism 9 software. The number of independent biological replicates is indicated as *n* in the figure legends. ANOVA *F*‐test was used to check homogeneity of variance, and Shapiro–Wilk test was used to check normality before unpaired *t*‐test using GraphPad Prism 9 software. A *p* value of less than .05 was considered statistically significant.

Additional methodological details are included in the Supplemental Materials.

## 3 RESULTS

### 3.1 Myocardial deletion of BSCL2 induces systolic heart dysfunction with dilation

Previously, we demonstrated lipodystrophic *Bscl2* **^−/−^** mice develop mild hypertrophic cadiomyopathy.^35^ In order to interrogate the specific role of cardiac BSCL2, we generated a mouse model with a cardiomyocyte‐specific deletion of BSCL2 (*Bscl2^cKO^*) via Myh6‐Cre. Gene expression analysis confirmed an approximate 75% reduction of *Bscl2* specifically in cardiac muscle but not in liver, skeletal muscle and epididymal white adipose tissue (eWAT) of *Bscl2^cKO^* mice (Figure S1(A)). Cardiac‐specific deletion of *Bscl2* also resulted in no change in body fat and lean masses (Figure S1(B)). We were not able to confirm the cardiac deletion of BSCL2 at protein level as no antibody is sensitive enough to detect the low‐abundance endogenous BSCL2 protein in murine hearts.

**FIGURE 1:**
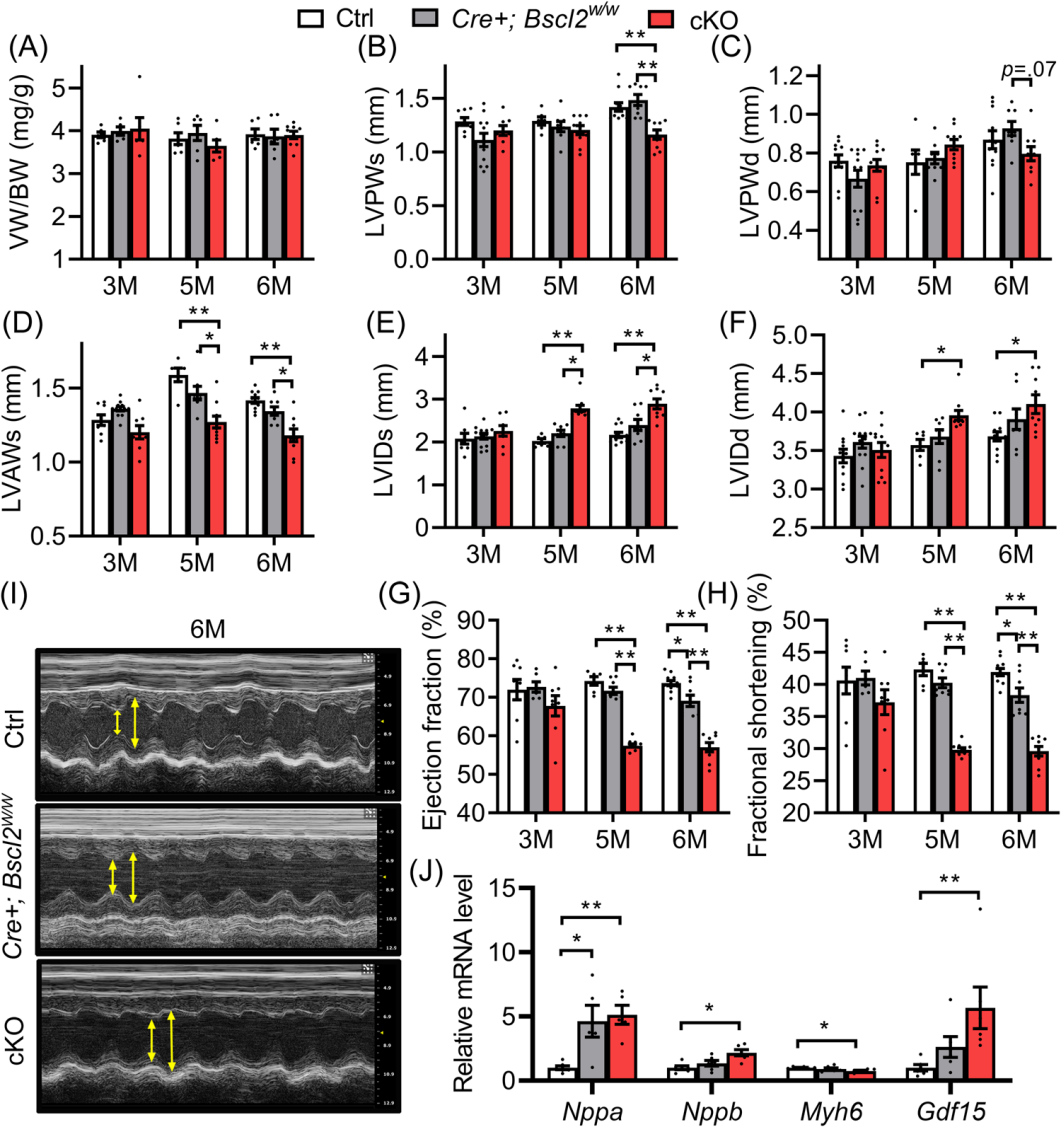
Mice with cardiac‐specific deletion of BSCL2 develop systolic heart dysfunction with dilation. (A) Ventricle weight (VW) normalised to body weight (BW) in 3‐month‐old (3 M), 5‐month‐old (5 M) and 6‐month‐old (6 M) male *Cre‐*;*Bscl2^f/f^* (Ctrl), *Cre+*;*Bscl2^w/w^*, and *Cre+*;*Bscl2^f/f^* (cKO) mice. 3 M old: Ctrl, *n *= 6; *Cre+*;*Bscl2^w/w^*, *n *= 5; cKO, *n *= 6. 5 M old: Ctrl, *n *= 6; *Cre+*;*Bscl2^w/w^*, *n *= 6; cKO, *n *= 6. 6 M old: Ctrl, *n *= 6; *Cre+*;*Bscl2^w/w^*, *n *= 6; cKO, *n *= 9. (B and C) left ventricle post wall thickness at end systole (LVPWs, mm) and at end diastole (LVPWd, mm), respectively; (D) left ventricle anterior wall thickness at end systole (LVAWs, mm); (E and F) left ventricle internal diameter at end systole (LVIDs, mm) or end diastole (LVIDd, mm); (G) ejection fraction (%) and (H) fractional shortening (%) in 3 M, 5 M and 6 M old male mice. 3 M old: Ctrl, *n *= 8; *Cre+*;*Bscl2^w/w^*, *n *= 8; cKO, *n *= 12. 5 M old: Ctrl, *n *= 6; *Cre+*;*Bscl2^w/w^*, *n *= 8; cKO, *n *= 10. 6 M old: Ctrl, *n *= 11; *Cre+*;*Bscl2^w/w^*, *n *= 8; cKO, *n *= 9. (I) Representative echocardiography, and (J) qRT‐PCR analysis of cardiac stress genes in ventricles of 6‐month‐old male mice. *n *= 6 per group. *, *p *< .05; **, *p *< .005. Two‐way ANOVA followed by Tukey's post‐hoc tests

We next determined the impact of cardiomyocyte‐specific deletion of *Bscl2* on cardiac function, in comparison with two distinct control groups [Ctrl and *Cre+;Bscl2^w/w^*]. No changes were observed in total body weights (Table S2), ventricle mass in proportion to body weight (Figure 1(A)) or tibia length (Figure S1(C)) at either 3 or 6 months of age between experimental groups. Three‐month‐old mice also did not display noticeable changes in left ventricular functions between the three experimental groups (Figures 1(B)–1(H)). Six‐month‐old *Cre+,Bscl2^w/w^* mice demonstrated no differences in left ventricle posterior wall thickness at systole (LVPWs) or diastole (LVPWd) (Figures 1(B) and 1(C)), left ventricle anterior wall thickness at systole (LVAWs) (Figure 1(D)) or diastole (LVAWd) (Table S2) and left ventricle internal diameter at systole (LVIDs) or diastole (LVIDd) (Figures 1(E) and 1(F)). However, they exhibited a minor reduction in ejection fraction and fractional shortening compared with Ctrl mice (Figures 1(G) and 1(H)), in agreement with a previous report.^40^ In contrast, 6‐month‐old *Bscl2^cKO^* mice exhibited decreased wall thickness, especially at systole (Figures 1(B)–1(D)) and increased dilation at both systoles and diastoles (Figures 1(E), 1(F), and 1(I)), along with marked reductions in contractile function compared with Ctrl and *Cre+,Bscl2^w/w^* mice (Figures 1(G) and 1(H)). To further pinpoint a cell‐autonomous role of BSCL2 in mediating cardiac function, we also compared echocardiography in 5‐month‐old mice, an age when the cardiac function of *Myh6‐Cre+* (*Cre+,Bscl2^w/w^*) mice is not compromised due to prolonged Cre expression. Not surprisingly, while the wall thicknesses of 5‐month‐old *Bscl2^cKO^* mice were marginally reduced, these mice still demonstrated significantly reduced contractile function accompanied with increased dilation compared to both Ctrl and *Cre+, Bscl2^w/w^* mice (Figures 1(B)–1(H)). In addition, we observed a greater induction of brain natriuretic peptide (*Nppb*) and growth differentiation factor 15 (*Gdf15*), and reduction of adult *Myh6* gene expression, in 6‐month‐old *Bscl2^cKO^* hearts versus Ctrl hearts (Figure 1(J)). The BSCL2 deletion‐induced cardiac dysfunction was not accompanied by abnormal cardiomyocyte morphology and excessive myocardial fibrosis (assessed by trichrome staining) in hearts of *Bscl2^cKO^* mice (Figures S1(D) and S1(E)). Together, these data suggest that cardiomyocyte‐specific BSCL2 deletion leads to systolic heart dysfunction independent of the long‐term expression of transgene Myh6‐Cre.

### 3.2 Myocardial‐specific deletion of BSCL2 causes elevated TG turnover and FAO preceding functional decline

**FIGURE 2:**
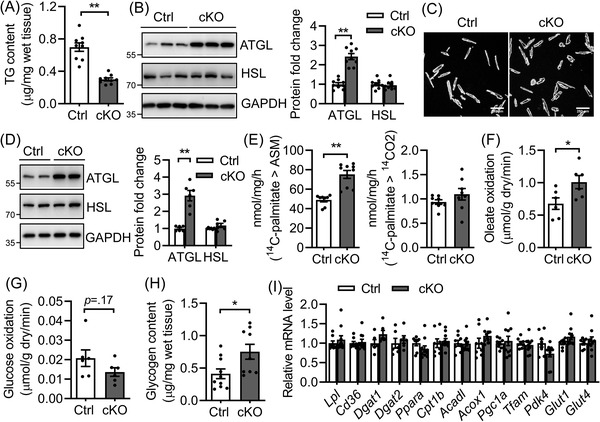
Cardiac‐specific deletion of BSCL2 induces cardiac triglyceride turnover and excessive fatty acid oxidation. (A) Ventricular TG content as normalised to wet tissue weight. *n *= 9 per group. (B) Representative Western blotting and quantification of lipolytic proteins in heart homogenates. *n *= 9 per group. (C and D) Isolated adult cardiomyocytes, representative Western blotting in duplicates and quantification from three independent experiments. (E) Acid soluble metabolites (ASM) and CO_2_ production after incubating heart crude mitochondrial fraction with ^14^C‐palmitate. Ctrl, *n *= 8; cKO, *n *= 9. (F) Oleate oxidation rate; (G) glucose oxidation rate in ex vivo perfused working hearts. *n *= 6 per group. (H) Glycogen content. Ctrl, *n *= 10; cKO, *n *= 9. (I) qRT‐PCR analysis of genes involved in fatty acid metabolism, mitochondrial biogenesis and glucose metabolism. Ctrl, *n *= 8; cKO, *n *= 10. All experiments used 3‐month‐old male *Cre−*;*Bscl2^f/f^* (Ctrl) and *Bscl2^cKO^* (cKO) mice. *, *p *< .05; **, *p *< .005, unpaired *t*‐tests (parametric)

It remains to be determined whether loss of BSCL2 in cardiomyocytes directly reduces cardiac TG contents as we have observed in lipodystrophic *Bscl2* **^−/−^** hearts.^35^ Indeed, we found reduced ventricular TG content (by 57%) (Figure 2(A)), increased protein expression of ATGL (≈2.5‐fold), but not HSL (Figure 2(B)), in 3‐month‐old *Bscl2^cKO^* mice compared with Ctrl mice. Changes in ATGL protein expression were not attributed to alterations in the mRNA level of *Pnpla2*, the gene encoding ATGL (Figure S2(A)). Isolated adult *Bscl2^cKO^* cardiomyocytes displayed similar upregulation of ATGL, but not HSL, compared with Ctrl cells (Figures 2(C) and 2(D)). Ctrl and *Bscl2^cKO^* cardiomyocytes responded similarly to the stimulation of isoproterenol in terms of PKA‐mediated phospholamban (PL) phosphorylation (Figure S2(B)). Analysis of whole heart lysates also revealed comparable basal PKA‐mediated substrate phosphorylation between Ctrl and *Bscl2^cKO^* mice (Figure S2(C)). These data suggest that cardiac‐specific deletion of BSCL2 did not affect cAMP/PKA signalling but induced posttranscriptional ATGL upregulation in hearts, different from what we previously observed in BSCL2‐deleted adipose tissue.^32^, ^33^, ^34^

Higher ATGL‐mediated lipolysis is coupled with cardiac FAO.^6^ When incubating heart homogenates with ^14^C‐palmitate, 3‐month‐old *Bscl2^cKO^* mice demonstrated elevated release of ^14^C‐labelled acid soluble metabolites despite a lack of change in ^14^CO_2_ production (Figure 2(E)). Ex vivo perfusions in working hearts from 3‐month‐old male Ctrl and *Bscl2^cKO^* mice identified no differences in heart rates, cardiac oxygen consumption, cardiac efficiency as well as cardiac power between two genotypes (Figures S2(D) and S2(G)), suggesting maintained cardiac function at this age. However, the *Bscl2^cKO^* hearts demonstrated a 33% increase in oleate oxidation (Figure 2(F)), concomitant with a tendency of lower glucose oxidation (Figure 2(G)) and an 83% increase of baseline glycogen content (Figure 2(H)). In spite of enhanced FAO and glycogen accumulation, *Bscl2^cKO^* hearts demonstrated no changes in the expression of genes involved in FA uptake (e.g., *Lpl*, *Cd36*), TG synthesis (e.g., *Dgat1*, *Dgat2*), mitochondrial and peroxisomal β‐oxidation (e.g., *Pparα*, *Cpt1β*, *Acadl, Acox1*), mitochondrial biogenesis (e.g., *Pgc1α*, *Tfam*) and glucose metabolism (e.g., *Pdk4*, *Glut1, Glut4*) (Figure 2(I)). Lack of changes in mitochondrial biogenesis was further confirmed by the similar ratios of mitochondrial DNA to nuclear DNA (Figure S2(H)) and protein levels of each of the electron transport chain (ETC) complexes between heart lysates of 3‐month‐old Ctrl and *Bscl2^cKO^* mice (Figure S2(I)). Together, these data clearly suggest that cardiomyocyte‐specific BSCL2 deficiency results in higher rates of cardiac TG turnover and FAO independent of transcriptional changes of mitochondrial and extra‐mitochondrial metabolic genes.

### 3.3 Chronic derangements in myocardial FAO leads to massive lipid remodelling and reduced endogenous substrates in *Bscl2^cKO^* hearts

**FIGURE 3:**
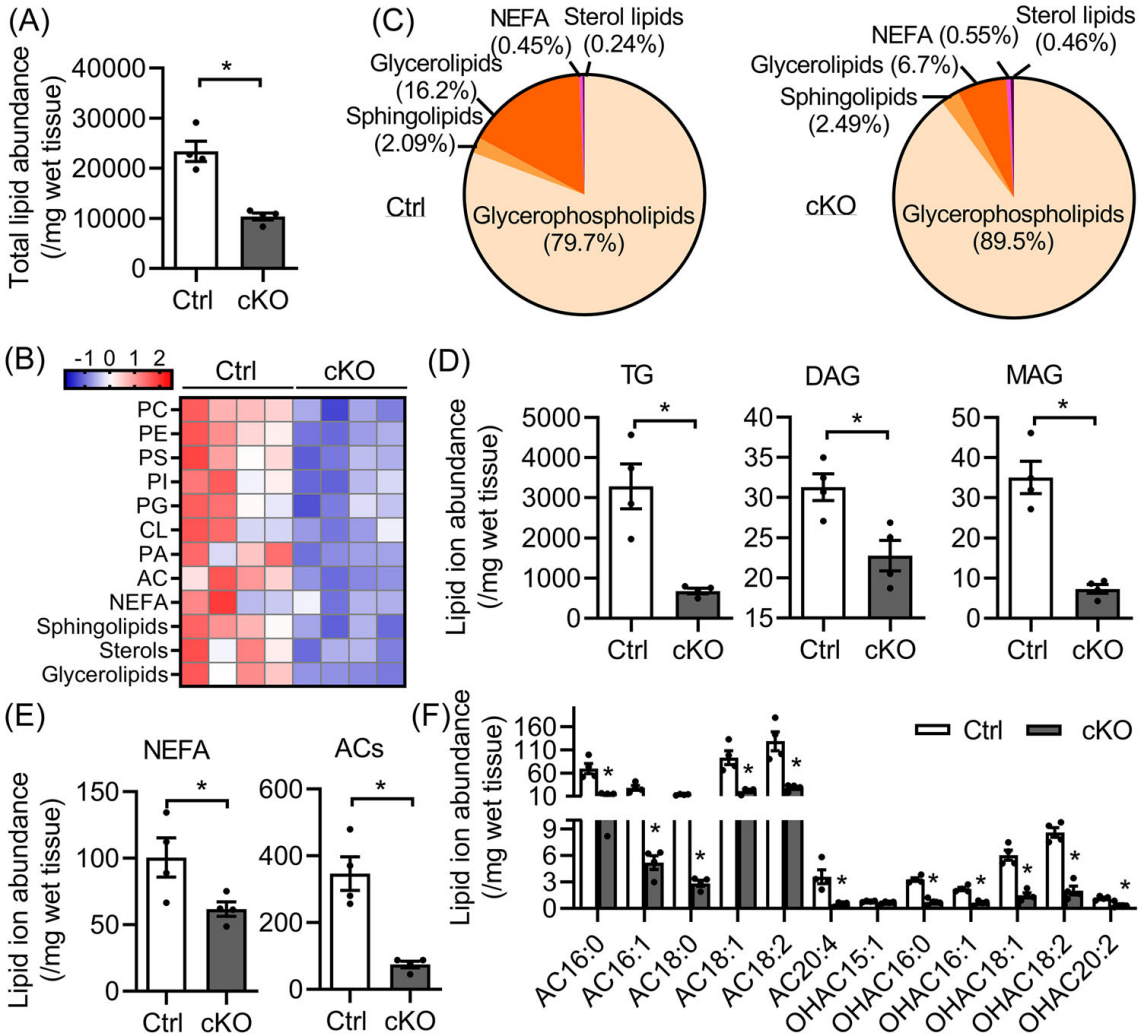
*Bscl2^cKO^* mice develop massive cardiac lipid remodelling and exhibit reduced metabolic reserve. (A) The total lipid ion abundance normalised to tissue weight. (B) Heatmap of lipid species including glycerophospholipids [i.e., phosphatidylcholine (PC), phosphatidylethanolamine (PE), phosphatidylserine (PS), phosphatidylinositol (PI), phosphatidylglycerol (PG), cardiolipin (CL), phosphatidic acid (PA) and acylcarnitines (AC)], non‐esterified free fatty acids (NEFA), sphingolipids, sterols and glycerolipids based on *Z*‐scores calculated from the summed ion abundances normalised to tissue weight. (C) Pie chart representing the proportional (%) distribution of summed ion abundances of glycerolipid, glycerophospholipid, sphingolipid, NEFA and sterol lipid classes. (D–F) Comparison of the total normalised ion abundances for (D) glycerolipids including TG, DAG and MAG, (E) NEFA and ACs, and (F) specific AC and hydroxyl acylcarnitines (OHAC) species. Global lipidomic analysis of ventricles by shotgun mass spectrometry was performed in non‐fasting 6‐month‐old male *Cre−*;*Bscl2^f/f^* (Ctrl) and *Bscl2^cKO^* (cKO) mice. *n *= 4 per group with each pooled from three animals. *, *p *< .05; **, *p *< .005, unpaired *t*‐tests (nonparametric)

To identify mechanisms underlying the development of HF, we performed untargeted lipidomic analyses of ventricles from 6‐month‐old Ctrl and *Bscl2^cKO^* mice. Total normalised lipid ion abundances identified in *Bscl2^cKO^* hearts were reduced by about 55% (Figure 3(A)). Heatmap analysis revealed massive reductions in the absolute levels of five broadly classified lipid classes defined by the Lipid MAPS consortium; that is, glycerophospholipids, fatty acyls [mainly non‐esterified fatty acids (NEFA)], sphingolipids, sterol lipids and glycerolipids in *Bscl2^cKO^* mice (Figure 3(B)). When comparing the % distributions of these five lipid classes, the proportions of glycerophospholipids, sphingolipids and sterol lipids were significantly higher in *Bscl2^cKO^* hearts compared with Ctrl hearts (Figure 3(C)). The proportions of NEFA were relatively comparable between two genotypes, whereas the proportion of glycerolipids was markedly reduced by 58% in *Bscl2^cKO^* hearts (Figure 3(C)). Analysis of the absolute levels of glycerolipids revealed 78%, 50% and 75% reductions in TG, diacylglycerol (DAG) and monoacylglycerol (MAG), respectively, in *Bscl2^cKO^* hearts (Figure 3(D)). The absolute levels of NEFA and total acylcarnitines (ACs) were significantly lower in *Bscl2^cKO^* hearts (Figure 3(E)). Specifically, the abundances of the major long‐chain ACs (AC16:0, AC16:1, AC18:0, AC18:1, AC18:2) were all reduced by approximately 70% (Figure 3(F)). These data suggest myocardial BSCL2 deletion results in a dramatic remodelling of lipid compositions highlighted by reduced levels of energy providing substrates indicative of impairment of cardiac metabolic reserve. Furthermore, only *Bscl2^cKO^* hearts displayed reduced cardiac TG contents (Figure S3(A)) and increased ATGL expression (Figure S3(B)), and no differences were observed between Ctrl and *Cre+;Bscl2^w/w^* hearts. The expression of PPARα and its target proteins (CD36, CPT1β) were also similar between three genotypes (Figure S3(B)). Collectively, these data emphasise a BSCL2‐specific regulation of lipid remodelling in *Bscl2^cKO^* hearts independent of transcriptional activation of PPARα.

### 3.4 Cardiomyopathy in aged *Bscl2^cKO^* mice is associated with energy deficiency independent of overt mitochondrial dysfunction and oxidative stress

**FIGURE 4:**
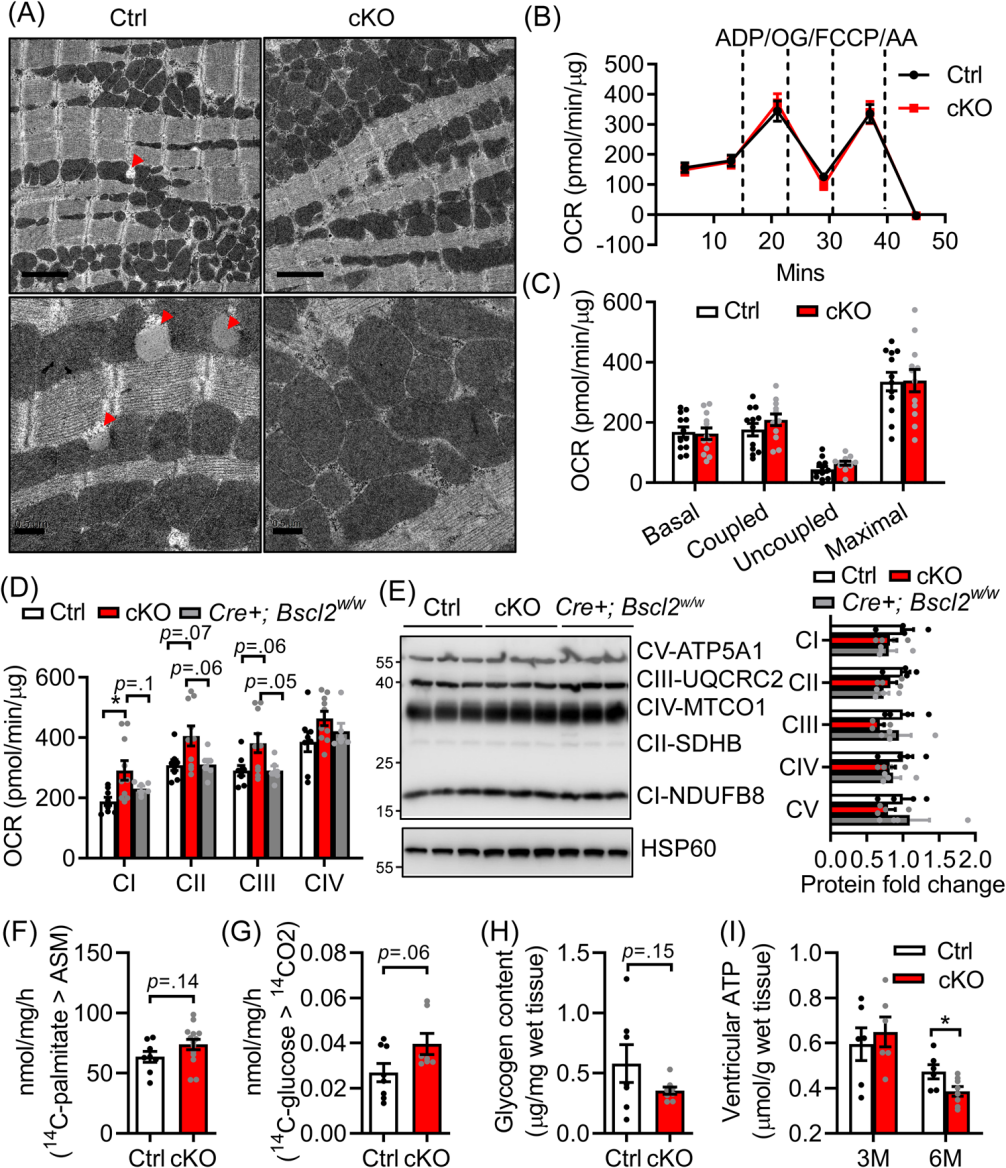
Cardiac dysfunction in *Bscl2^cKO^* mice is associated with bioenergetics failure independent of mitochondrial respiratory dysfunction. (A) Representative transmission electron micrographs of ventricles from 6‐month‐old *Cre−*;*Bscl2^f/f^* (Ctrl) and *Bscl2^cKO^* (cKO) mice. Red arrowheads: lipid droplets (LDs). Upper panels: scale bar = 2 μm; lower panels: scale bar = 0.5 μm. *n* = 3 per group. (B) Succinate/rotenone‐driven mitochondrial oxygen consumption rates (OCR) were measured in isolated mitochondria from hearts of 6‐month‐old Ctrl and cKO mice by Seahorse XF24 analyser with sequential addition of ADP, oligomycin (OG), FCCP and antimycin A (AA). Basal, coupled, uncoupled and maximal mitochondrial respiration were shown in (C). *n *= 4 in triplicates per group. Two‐way ANOVA followed by Tukey's post‐hoc tests. (D) Electron flow assays measuring complex I–IV activities (*n* = 3 in triplicates) and (E) representative Western blotting and quantification in isolated mitochondria from ventricles of 6‐month‐old Ctrl, *Bscl2^cKO^*, *Cre+;Bscl2^w/w^* mice. *n *= 4 per group. Mitochondrial HSP60 was used as loading control. (F) Acid‐soluble metabolites (ASM) after incubating heart crude mitochondrial fraction with ^14^C‐palmitate. Ctrl, *n *= 4 in duplicates; cKO, *n *= 6 in duplicates. (G) Glucose oxidation rate after incubating heart crude mitochondrial fraction with ^14^C‐glucose. *n *= 6 per group. (H) Ventricular glycogen contents, *n *= 7 per group. Panels F and G were performed in 6‐month‐old Ctrl and cKO mice. Unpaired *t*‐tests (parametric). (I) ventricular ATP contents in non‐fasting 3 and 6‐month‐old male *Ctrl* and *Bscl2^cKO^* mice. 3 M: Ctrl, *n *= 6; cKO, *n *= 7, 6 M: Ctrl, *n *= 6; cKO, *n *= 8. *, *p *< .05, multiple unpaired *t*‐tests with Holm–Sidak method

We next examined whether the massive lipid remodelling exerts maladaptive effects on mitochondrial function, leading to the progressive development of HF when *Bscl2^cKO^* mice age. Transmission electron microscopy images revealed a complete lack of LDs in 6‐month‐old *Bscl2^cKO^* hearts, in support of cardiac TG reduction (Figure 4(A)). Sarcomere arrangement, mitochondrial morphology and sizes, as well as mitochondria distribution were generally preserved in both Ctrl and *Bscl2^cKO^* hearts (Figure 4(A)). Respirational analysis of isolated mitochondria from hearts of Ctrl and *Bscl2^cKO^* mice identified similar basal (in the presence of exogenous succinate and rotenone) and maximal oxygen consumption rates, as well as coupled (ADP‐driven, Complex V) and uncoupled respiration (Figures 4(B)–4(C)). Interestingly, the activity of Complex I (CI) was significantly increased, whereas the activities of CII and CIII trended higher in *Bscl2^cKO^* mitochondria compared with those from Ctrl or *Cre+;Bscl2^w/w^* mice (Figure 4(D)). Again, the expression of marker proteins per mitochondrial mass for each mitochondrial ETC complex remained comparable (Figure 4(E)), which was also the case in the whole hearts of 6‐month‐old *Bscl2^cKO^* mice (Figure S4(A)). There were no differences in the expressions of PGC1α and mitochondrial stress and uncoupling proteins (Prohibitin, UCP2 and UCP3) in *Bscl2^cKO^* hearts compared to both Ctrl and *Cre+;Bscl2^w/w^* hearts (Figure S4(A)). Six‐month‐old *Bscl2^cKO^* hearts still possessed a tendency of higher capacity to oxidise exogenous palmitate relative to Ctrl hearts (Figure 4(F)); while its glucose oxidation rate trended higher (Figure 4(G)), resulting in a tendency toward lower cardiac glycogen content in these mice (Figure 4(H)). Oxidation of 2′, 7′‐dichlorofluorescein diacetate was not augmented in heart extracts of 6‐month‐old *Bscl2^cKO^* mice (Figure S4(B)). The level of lipid peroxides malondialdehyde was even reduced in *Bscl2^cKO^* hearts compared with Ctrl hearts (Figure S4(C)). Consistent with lack of oxidative stress, there were no differences in the expression of SOD1, SOD2 and Catalase in *Bscl2^cKO^* hearts relative to both Ctrl and *Cre+;Bscl2^w/w^* hearts (Figure S4(A)). Furthermore, genes regulating glutathione metabolism (e.g., *Gclc*, *Gsr*, *Gss*) were largely unaltered although GPX3 expression showed a trend toward increase in both *Cre+;Bscl2^w/w^* and *Bscl2^cKO^* hearts compared with Ctrl hearts (Figure S4(D)). These data suggest that mitochondrial dysfunction and oxidative stress are unlikely to play roles in maladaptive cardiac remodelling and progression of HF in *Bscl2^cKO^* mice.

While the ventricular ATP content was maintained in 3‐month‐old *Bscl2^cKO^* mice, it was significantly lower when *Bscl2^cKO^* mice reached 6‐month‐old (Figure 4(I)), in line with the reduced cardiac metabolic reserve (i.e., lipid and glycogen stores). Furthermore, there were no differences in the plasma concentrations of glucose and lipid substrates (TG, cholesterol, NEFA and glycerol) between 6‐month‐old Ctrl and *Bscl2^cKO^* mice (Table S3). These data suggest that deprivation of myocellular endogenous substrates is likely responsible for the progressive metabolic transition leading to cardiac energetic and contractile failure in *Bscl2^cKO^* mice.

### 3.5 Inhibition of cardiac TG lipolysis or FAO partially rescues cardiac dysfunction in *Bscl2^cKO^* mice

Since cardiac dysfunction in *Bscl2^cKO^* mice is associated with higher ATGL expression and FAO, we first generated mice with partial loss of ATGL in *Bscl2^cKO^* mice (*A^h^B^cKO^*) (Figure S5(A)). ATGL haploinsufficiency indeed abolished cardiac ATGL upregulation in *B^cKO^* mice, with only 50% myocardial ATGL present compared with Ctrl hearts (Figure S5(B)). *A^h^B^cKO^* mice showed no alterations in body weights, and VW to TL ratios when compared with Ctrl and *B^cKO^* mice (Figure S5(C) and Table S4). Partial ATGL loss neither significantly restored the wall thicknesses nor reduced dilation of *B^cKO^* mice (Figure S5(D) and Table S4). However, the cardiac contractile function was markedly improved in *A^h^B^cKO^* mice when compared with *B^cKO^* mice, although still impaired relative to Ctrl mice (Figures S5(E) and S5(F), respectively). Partial deletion of ATGL led to a minimal elevation of ventricular TG accumulation (Figure S5(G)). These data suggest cardiac ATGL upregulation indeed contributes to the development of cardiac dysfunction in *Bscl2^cKO^* mice.

**FIGURE 5:**
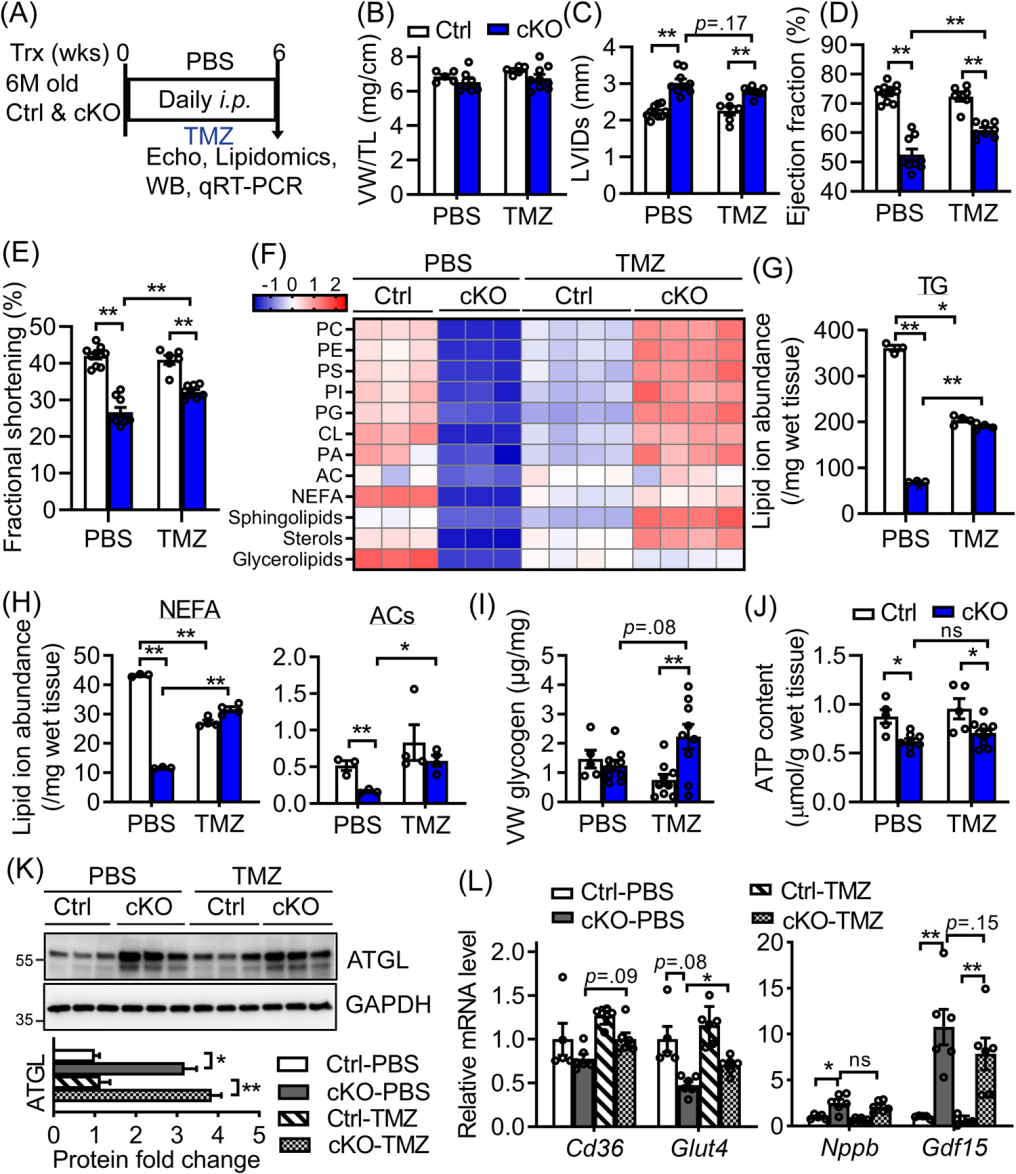
Inhibition of fatty acid oxidation partially improves metabolic reserve and cardiac function in *Bscl2^cKO^* mice. (A) 6‐month‐old male *Cre−*;*Bscl2^f/f^* (Ctrl) and *Bscl2^cKO^* (cKO) mice received daily i.p. injection with PBS or trimetazidine (TMZ) at 15 mg/kg body weights (BW) for a total of 6 weeks. (B) Ratio of ventricle weight (VW) to tibia length (TL). Ctrl, *n *= 5; cKO, *n *= 9. (C) Left ventricular internal diameter in end systole (LVIDs); (D) ejection fraction and (E) fractional shortening. PBS‐Ctrl, *n *= 10; PBS‐cKO, *n *= 9. *n *= 9 per TMZ‐treated group. (F) Heatmap of major lipid species based on *Z*‐score calculated from the summed ion abundances normalised to milligram of wet tissue; (G and H) comparison of the total normalised ion abundances for (G) glycerolipids as well as (H) NEFA and ACs. *n *= 3 per PBS‐treated group. *n *= 4 per TMZ‐treated group. Each sample was pooled from three animals. (I) Ventricular glycogen. PBS‐Ctrl, *n *= 5; PBS‐cKO, *n *= 9. *n *= 9 per TMZ‐treated group. (J) Ventricular ATP contents normalised to gram of wet tissue. PBS‐Ctrl, *n *= 5; PBS‐cKO, *n *= 8. TMZ‐Ctrl, *n *= 5, TMZ‐cKO, *n *= 9. (K) Representative Western blotting and quantification of total cell extracts from ventricles of 6‐month‐old PBS and TMZ‐treated Ctrl and *Bscl2^cKO^* mice. *n *= 6 per group. Data were normalised to GAPDH with PBS‐treated Ctrl set to 1. Two independent experiments. (L) qRT‐PCR analysis of lipid and glucose transport genes and cardiac stress genes in ventricles of 6‐month‐old PBS and TMZ‐treated male mice. *n *= 6 per group. *, *p *< .05; **, *p *< .005. ns: not significant. Two‐way ANOVA followed by Tukey's post‐hoc tests

To further dissect whether elevated FAO is involved in the pathogenesis of HF in *Bscl2^cKO^* mice, we treated mice with TMZ, a specific 3‐Ketoacyl‐CoA thiolase (3‐KAT) inhibitor that inhibits FAO,^37^ for up to 6 weeks starting at 6‐month‐old when *Bscl2^cKO^* mice already developed cardiac dysfunction (Figure 5(A)). TMZ did not alter body weight (Figure S6(A)) nor liver weight (Figure S6(B)), but tended to increase white fat mass in both genotypes (Figure S6(C)). TMZ elevated circulating cholesterol levels in both Ctrl and *Bscl2^cKO^* mice, but there were no differences in plasma glucose, NEFA, glycerol or TG levels in the experimental groups (Table S5). Ctrl and *Bscl2^cKO^* mice also demonstrated similar ventricle to body weight (Figure S6(D)) and tibia length (Figure 5(B)) ratios after TMZ treatment. TMZ produced no changes in cardiac dilation and contractile function of Ctrl mice. However, it was able to significantly improve LVPWs and cardiac contractile function (including increasing fractional shortening by 20.6%) without notably normalising dilatation in *Bscl2^cKO^* hearts (Figures 5(C)–5(E) and Table S5). Attenuation of cardiac dysfunction by TMZ was also evident in female *Bscl2^cKO^* mice (Figure S6(E)–S6(H)).

We performed another independent set of untargeted lipidomics to examine the effect of TMZ on cardiac lipidome. As indicated in Figure 5(F), PBS‐treated *Bscl2^cKO^* hearts recapitulated all changes in cardiac lipid contents as we previously observed in 6‐month‐old *Bscl2^cKO^* hearts (Figure 3). TMZ unexpectedly reduced the absolute abundances of cardiac lipids in all categories except ACs in Ctrl hearts. In contrast, TMZ dramatically enhanced the accumulation of these lipid classes in *Bscl2^cKO^* hearts relative to PBS‐treated *Bscl2^cKO^* hearts. Especially, the absolute levels of phospholipids, sphingolipids, and sterols were even greater in TMZ‐treated *Bscl2^cKO^* than PBS‐treated Ctrl hearts (Figure 5(F)). Specifically, TMZ markedly increased TG, NEFAs and ACs in *Bscl2^cKO^* hearts (Figures 5(G) and 5(H)). While it slightly lowered glycogen in Ctrl hearts, it caused more glycogen accumulation in *Bscl2^cKO^* hearts (Figure 5(I)). Despite a significant upregulation of metabolic reserve, there was only a very minimal but non‐significant improvement of ventricular ATP content in TMZ‐treated *Bscl2^cKO^* hearts (Figure 5(J)). As expected, TMZ itself did not abolish ATGL upregulation in *Bscl2^cKO^* hearts, since it acts on FAO downstream of ATGL (Figure 5(K)). Downregulation of lipid and glycogen contents in TMZ‐treated Ctrl heart could not be explained by similar expression levels of *Cd36* and *Glut4*, two key genes governing cardiac lipid and glucose entries. However, we did observe a slight upregulation of these two genes in TMZ‐treated compared to PBS‐treated *Bscl2^cKO^* hearts, in line with their elevated lipid and glycogen contents (Figure 5(L)). Furthermore, there was also a trend for TMZ to attenuate the gene expression of cardiac stress markers in *Bscl2^cKO^* hearts (Figure 5(L)). Together, our data suggest that inhibiting FAO in *Bscl2^cKO^* hearts is able to partially restore cardiac function, potentially through modifying cardiac metabolism.

### 3.6 Preventative treatment of HFD significantly attenuates cardiac bioenergetics deficiency and dysfunction in *Bscl2^cKO^* mice

**FIGURE 6:**
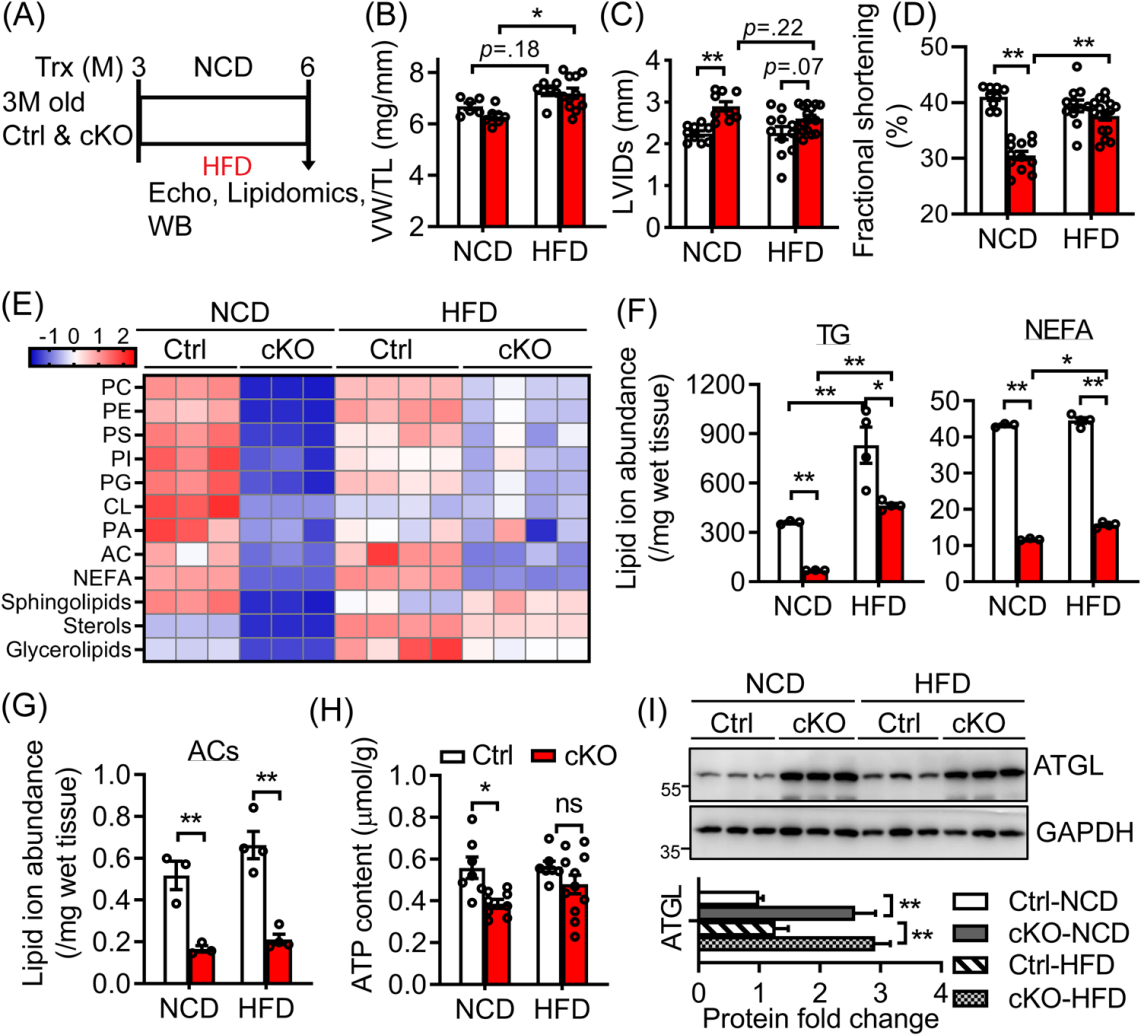
High‐fat diet feeding increases metabolic reserve and prevents cardiac dysfunction in *Bscl2^cKO^* mice. (A) 3‐month‐old *Bscl2^f/f^* (Ctrl) and *Bscl2^cKO^* (cKO) mice were fed with normal chow diet (NCD) or high‐fat diet (HFD, 60% fat calories) for a total of 3 months (M). (B) Ratio of ventricle weight (VW) to tibial length (TL). NCD‐Ctrl, *n *= 6; NCD‐cKO, *n *= 7; HFD‐Ctrl, *n *= 7; HFD‐cKO, *n *= 11. (C) Left ventricular internal diameter in end systole (LVIDs); and (D) fractional shortening. NCD‐Ctrl, *n *= 9; NCD‐cKO, *n *= 12; HFD‐Ctrl, *n *= 12; HFD‐cKO, *n *= 16. (E) Heatmap of major lipid species based on *Z*‐score calculated from summed ion abundances normalised to milligram of wet tissue; (F and G) comparison of the total normalised ion abundances for (F) glycerolipids and NEFA as well as (G) ACs. *n *= 3 per NCD group. *n *= 4 per HFD group. Each sample was pooled from three animals. (H) Myocardial ATP content as normalised to gram of wet tissue. NCD‐Ctrl, *n *= 8; NCD‐cKO, *n *= 8; HFD‐Ctrl, *n *= 7; HFD‐cKO, *n *= 11. (I) Representative Western blotting of total heart extracts from ventricles of Ctrl and *Bscl2^cKO^* mice. *n *= 3 per group. Two independent experiments. *, *p *< .05; **, *p *< .005. Two‐way ANOVA followed by Tukey's post‐hoc tests

To gain further insight into the importance of metabolic reserve in energy‐starved *Bscl2^cKO^* hearts, we also fed 3‐month‐old Ctrl and *Bscl2^cKO^* male mice a normal chow diet (NCD) or a 60% HFD for a period of 3 months (Figure 6(A)). By 6 months old, Ctrl and *Bscl2^cKO^* mice exhibited similar obese phenotype with comparable weight gains after HFD feeding (Table S3). They exhibited similarly higher levels of plasma glucose, cholesterol, NEFA and glycerol relative to their NCD‐fed counterparts, and there were no differences in serum TG concentrations in all four groups (Table S3). HFD increased comparable cardiac hypertrophy as evidenced by ventricle weight to tibia length ratios and a tendency of increase in wall thickness in both genotypes (Figure 6(B) and Table S3). There were no significant changes in dilation and contractile function in Ctrl mice after 3 months of HFD feeding (Figures 6(C) and 6(D) and Table S3). However, HFD was able to increase wall thickness (LVPWs) and improve cardiac contractile function in *Bscl2^cKO^* mice to the levels similar to HFD‐fed Ctrl mice despite exerting no effect on dilation (Figures 6(C) and 6(D) and Table S3). Interestingly, in Ctrl hearts, we found HFD reduced the amounts of phospholipids (i.e., PC, PS, PI, PG, CL and PA) and sphingolipids, but preferentially increased the levels of sterols and glycerolipids when compared with NCD (Figure 6(E)). The amounts of almost all lipid classes were increased in *Bscl2^cKO^* hearts after HFD feeding, albeit still lower than HFD‐fed Ctrl hearts (Figure 6(E)). Specifically, TG levels were greatly upregulated in both Ctrl and *Bscl2^cKO^* hearts after HFD feeding, whereas the levels of NEFA and ACs in *Bscl2^cKO^* hearts were increased to a lesser extent by HFD when compared with NCD (Figures 6(F) and 6(G)). Nevertheless, while *Bscl2^cKO^* hearts contained less ATP under NCD, the ventricular ATP contents in HFD‐fed *Bscl2^cKO^* mice was partially recovered to the levels of HFD‐fed Ctrl hearts, suggesting improved cardiac energetics (Figure 6(H)). Immunoblot analysis revealed similar upregulation of ATGL in *Bscl2^cKO^* relative to Ctrl hearts regardless of diets (Figure 6(I)). Collectively, HFD could restore cardiac function by improving cardiac energetics via increasing energy supply in *Bscl2^cKO^* mice.

## 4 DISCUSSION

**FIGURE 7:**
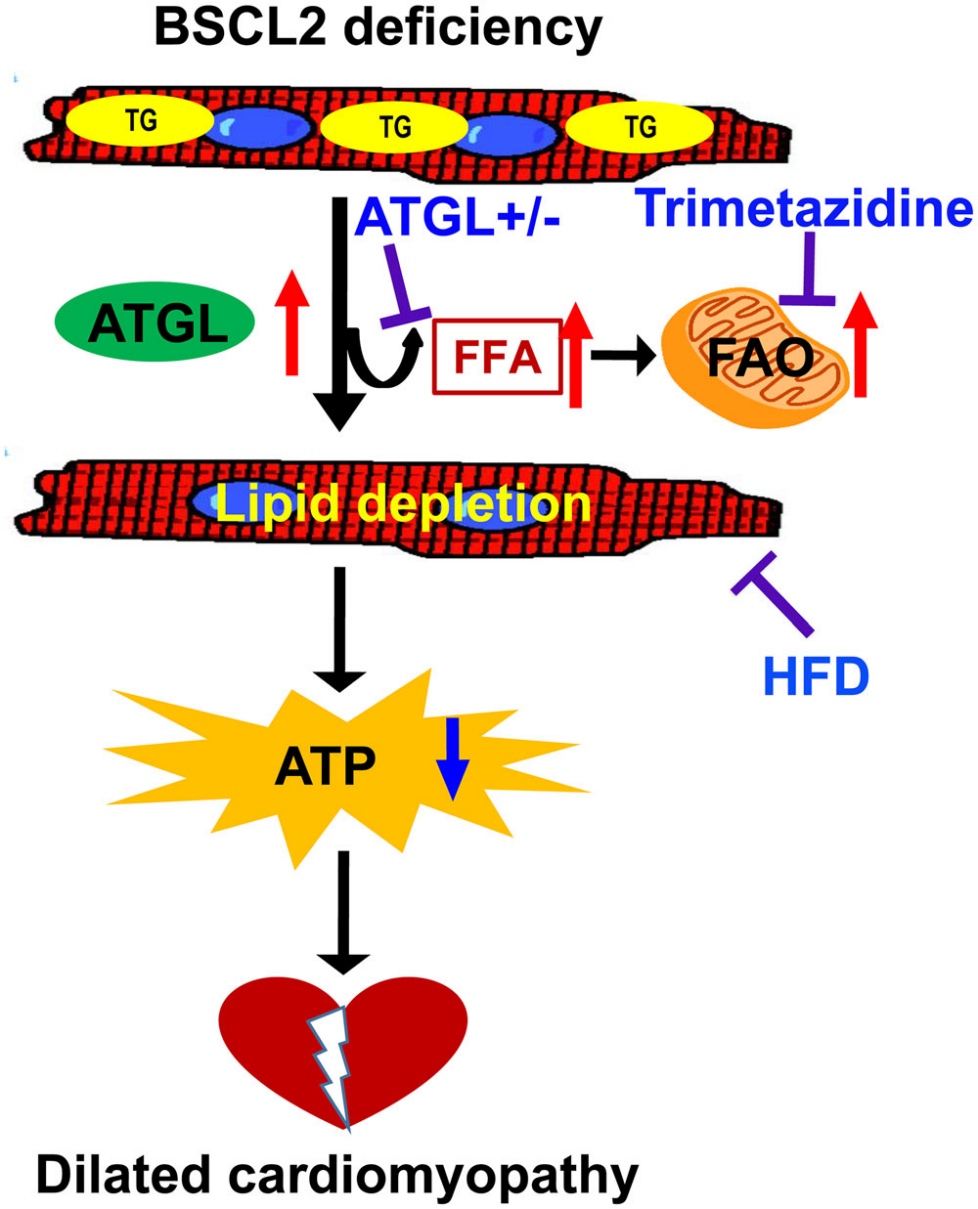
Schematic diagram of the indispensable role of BSCL2 in regulating cardiac lipid metabolism and function. Cardiac deletion of BSCL2 causes ATGL overexpression and excessive fatty acid oxidation (FAO) which exhaust intramyocellular triglyceride (TG) and induce drastic depletion of cardiac lipidome, ultimately resulting in energetic and contractile failure in mice. Partial deletion of ATGL, inhibiting FAO by trimetazidine or increasing lipid supply via high‐fat diet (HFD) feeding replenishes cardiac lipidome and alleviates cardiac dysfunction caused by loss of BSCL2

In this study, we show that genetic deletion of BSCL2 in cardiomyocytes leads to dramatic cardiac lipid remodelling and systolic heart dysfunction with dilation in mice. Mechanistically, cardiac BSCL2 ablation causes ATGL overexpression, excessive FAO and overt cardiac lipid remodelling. Increased lipid catabolism ultimately exhausts intramyocellular lipid and glycogen reserve and is likely responsible for the energetic and contractile dysfunction in *Bscl2^cKO^* mice. Importantly, inhibiting TG turnover by partial genetic deletion of ATGL or suppressing FAO by promoting substrate switch or HFD feeding via increasing lipid supply can partially mitigate cardiac dysfunction in *Bscl2^cKO^* mice (Figure 7). Our results thus identify a novel and indispensable role of BSCL2 in regulating a preferential oxidation of FAs from endogenous cardiac TG lipolysis which governs cardiac energetics and function.

BSCL2 deletion enhances cAMP/PKA triggered ATGL‐mediated lipolysis and FAO in adipose tissue.^32^, ^33^, ^34^ Similar to global *Bscl2* **^−/−^** hearts,^35^ *Bscl2^cKO^* hearts exhibited higher ATGL expression associated with reduced TG contents and accelerated FAO, highlighting the cell‐autonomous role of cardiac BSCL2 in lipid catabolism. Interestingly, lipodystrophic *Bscl2^−/−^* mice develop cardiac hypertrophy due to increased basal IGF1 receptor (IGF1R)‐mediated PI3K/AKT signalling secondary to hyperinsulinemia.^35^ These mice develop mild cardiomyopathy, which can be attenuated by partially restoring fat mass and/or improving whole‐body and cardiac insulin resistance.^35^, ^41^ This suggests that insulin resistance largely accounts for the pathophysiology of metabolic cardiomyopathy in lipodystrophic *Bscl2* **^−/−^** mice, resembling diabetic hearts. Indeed, in patients with congenital generalised lipodystrophy, leptin treatment, which is known to improve metabolic complications including insulin resistance, has been recently shown to attenuate cardiac hypertrophy and increase contractile function.^42^ On the other hand, *Bscl2^cKO^* mice exhibit neither left ventricular hypertrophy nor functional changes at 3 months old despite an increase in FAO. By 6 months old, these mice tend to have thinner left ventricular wall and develop energy deficit‐induced systolic dysfunction but not end‐stage HF. Such changes occur independent of profound structural remodelling (e.g., hypertrophy and fibrosis) (Figure 1) and insulin resistance in *Bscl2^cKO^* hearts (data not shown). Notably, a previous report identified no differences in heart weights of 1‐year‐old mice with cardiac deletion of BSCL2 driven by the same Myh6‐Cre.^43^ However, cardiac function was not directly assessed and it remains unknown whether BSCL2 directly controls cardiac function in their model.

Alleviation of cardiac dysfunction by partial ATGL loss in *Bscl2^cKO^* mice provides solid evidence that an uncontrolled ATGL‐mediated lipid catabolism plays a part in impairing cardiac performance (Figure S5). ATGL‐mediated fat catabolism has been directly linked to cardiac PGC‐1/PPARα expression and FAO rates.^6^ While *Bscl2^cKO^* hearts clearly exhibited excessive ATGL‐coupled FAO preceding impaired cardiac function, none of PGC‐1α and PPARα and their downstream target genes were altered (Figure 2). However, lean mice with myocardial ATGL overexpression^8, 9^ or acetyl‐CoA carboxylase 2 (ACC2) deletion^44^ maintain normal cardiac energetics and performance despite higher TG turnover or FAO rates. Thus, other changes in cellular metabolism independent of ATGL‐mediated intramyocardial lipid catabolism may exist to contribute to the cardiac energy deficit and progressive deterioration of cardiac function in *Bscl2^cKO^* mice at baseline, which warrants further investigation.

ATGL is known to be ubiquitinated (Ref. ^45^ and data not shown) and we have previously demonstrated enhanced ATGL stability in *Bscl2* **^−/−^** cardiomyocytes and mouse embryonic fibroblasts (MEFs).^35^ However, we were not able to pull down endogenous ATGL using current available antibodies, which prevents us from examining ATGL ubiquitination in BSCL2‐deleted hearts. Therefore, the molecular events for the posttranscriptional regulation of ATGL in the absence of BSCL2 remain to be identified.

Reliance on FAO in obesity and/or diabetes is correlated with lower cardiac efficiency, impaired mitochondrial respiratory function and increased ROS production.^46^ Alteration of the cardiac lipidome may also mediate functional impairment through dampening mitochondrial function.^47^ However, none of these abnormalities occurred in our *Bscl2^cKO^* mice despite massive reduction of cardiac lipid contents. In fact, mitochondrial function still trends higher even in BSCL2‐deleted hearts with reduced ATP levels. Such finding is seemingly counterintuitive, as it is hard to associate higher myocardial mitochondrial function with reduced ATP level. It is worth to mention that mitochondrial hyperactivation in *Bscl2^cKO^* hearts was derived from in vitro or ex vivo assays performed in the presence of excessive exogenous substrates, whereas this may not be the case in vivo. Higher cardiac FAO is normally associated with increased exogenous lipid import as observed in diabetic hearts or hearts with cardiac‐restricted overexpression of PPARα.^48^ This was also not true with our *Bscl2^cKO^* mice which displayed similar expression of lipid uptake genes and comparable circulating lipid metabolites. Therefore, imbalance of lipid consumption and supply within *Bscl2^cKO^* myocardium leads to downregulation of the vast amounts of lipid substrates (Figure 3), contributing to the ATP deficit in *Bscl2^cKO^* hearts. In addition, AC levels tightly reflect the FAO rates, and AC profiling has been used to identify FAO dysregulation.^49^ Notably, *Bscl2^cKO^* hearts demonstrated no alterations in CPT1 expression, suggesting intact carnitine shuttle (Figure S3(B)). The almost unanimous reduction of long‐chain ACs in *Bscl2^cKO^* hearts highlights the presence of increased FAO which may eventually deplete mitochondrial ACs thus reducing substrates entering TCA cycles and causing energy deficit. While it is plausible that BSCL2 deletion is still causing constant hydrolysis of the remaining TGs and channelling FAs to mitochondria for combustion in a compartmentalised domain in vivo, the energy status of the whole myocardium is ultimately compromised due to the limited availability of substrates. One limitation of our study is that the amount of myocardial ATP was not directly measured by NMR and probably does not reflect the whole myocardium ATP reserve. Nevertheless, our *Bscl2^cKO^* mice constitute as the first animal model that demonstrates excessive myocardial lipid catabolism associated with deterioration of metabolic reserve and cardiac dysfunction in the absence of elevated lipid uptake.

Treatment with TMZ results in an insignificant increase of skeletal muscle TG content and higher plasma AC in HFD‐induced obese mice.^37^ However, thus far, no study has examined the effects of TMZ treatment on myocardial lipid and glycogen contents. Surprisingly, we identified TMZ drastically remodels cardiac lipidome by downregulating the lipid contents of almost all lipid classes in normal mouse hearts. Whether this is primary to the cardiac effect of TMZ remains unknown. TMZ improves cardiac function in ischemia/reperfusion injury predominantly by shifting energy production from NEFA to glucose oxidation in the heart.^50^, ^51^, ^52^, ^53^ The mechanisms underlying the cardioprotective role of TMZ in our *Bscl2^cKO^* mice could not be simply explained by alleviation of energy deficit, as TMZ failed to significantly improve intracellular ATP levels in *Bscl2^cKO^* hearts. Conversely, TMZ promotes drastic lipid and glycogen accumulation in metabolic‐stressed *Bscl2^cKO^* hearts. The prominence of glycogen in TMZ‐treated *Bscl2^cKO^* hearts may reflect enhanced glucose utilisation as glycogen content has also been shown to commensurate with augmented carbohydrate metabolism such as in GLUT1‐overexpressing hearts.^54^ Interestingly, aside from glucose utilisation, TMZ has been recently shown to induce cardiac β‐hydroxybutyrate flux to attenuate isopropanol‐induced rat HF.^55^ Whether TMZ indeed induces substrate switch to glucose and ketone bodies in *Bscl2^cKO^* hearts needs to be further dissected by ex vivo heart perfusion experiments. Meanwhile, it is not clear whether TMZ treatment benefits the chronically dysfunctional myocardium by metabolically acting on heart itself or other organs such as liver, skeletal muscle and adipose tissue to ultimately moderate cardiac substrate utilisation. In addition, whether TMZ inhibits cardiac FAO in vivo still remains controversial,^56^ and we also cannot exclude the non‐metabolic effects of TMZ in preventing left ventricle remodelling independent of its inhibitory activity on FAO.^57^, ^58^ Nevertheless, our study underscores that TMZ has potential in ameliorating cardiac function and slows HF progression in a non‐ischemic model of HF.

HFD alone has been shown to be cardioprotective especially in alleviating energy‐compromised HF.^59^ In the present study, we find that decreased cardiac function in *Bscl2^cKO^* mice can be attenuated by HFD feeding. Lipidomics study further confirmed improved cardiac metabolic substrates mainly in the form of TG in HFD‐fed *Bscl2^cKO^* mice. This was in line with the notion that HFD feeding provides more coronary circulation of substrates to match up the rate of enhanced lipid utilisation thus attenuating the myocardial ATP deficit in the energy‐deprived failing *Bscl2^cKO^* hearts. In spite of an improvement in cardiac contractile function, we were not able to observe a significant reduction of cardiac stress markers in HFD‐treated *Bscl2^cKO^* mice (not shown). Nonetheless, results from our HFD feeding studies support the cardio‐protective effect of HFD on the energy‐deprived HF.

## 5 CONCLUSIONS

Our study highlights an important link between BSCL2 and myocardial energy metabolism and function and advances our understanding of the relationship between TG dynamics, FAO rates and the pathogenesis of HF. It may also provide insights into the therapeutic approaches in the treatment of cardiac disorders related to dysregulated lipid metabolism in general.

## CONFLICT OF INTEREST

None declared.

## CONSENT FOR PUBLICATION

Not applicable.

## Supporting information

Supporting information
