# Supplementary material for: BSCL2/Seipin deficiency in hearts causes cardiac energy deficit and dysfunction via inducing excessive lipid catabolism
Source: Clin Transl Med. 2022 Apr 5;12(4):e736. doi: 10.1002/ctm2.736 (PMC8982503; doi:10.1002/ctm2.736)
Supplement: Supplementary file 1 — Supporting information [file CTM2-12-e736-s001.docx]

**Supplemental Data**

**BSCL2/Seipin Deficiency in Heart Causes Energy Deficit and Heart Failure through Inducing Excessive Lipid Catabolism**

**Supplemental Materials and Methods**

**Histology, transmission electron microscopy (TEM), and trichrome staining**

Tissues were formalin fixed, paraffin embedded, sectioned and stained with Hematoxylin–eosin (H&E). Whole hearts were fixed, embedded and cut along the coronal plane to visualize the four-chamber view. The apex of left ventricle (LV) tissue was fixed and stained for electron microscopical imaging in a JEM 1230 transmission electron microscope (JEOL USA Inc., Peabody, MA) at 110 kV with an UltraScan 4000 CCD camera & First Light Digital Camera Controller (Gatan Inc., Pleasanton, CA) as previously described^1^. Trichrome staining was performed using Trichrome Staining Kit (Abcam, ab150686) according to manufacturers’ directions.

**Plasma biochemistry**

Blood glucose levels were measured by One-touch Ultra glucose meter. Glycerol and NEFA levels were determined using a free glycerol reagent (Sigma-Aldrich) and WAKO NEFA analysis kit (NEFA-HR(2); Wako Pure Chemical Industries), respectively. Plasma triglyceride and cholesterol levels were measured by colorimetrical analyses using triglyceride assay kit (Infinity^TM^ triglycerides kit, Thermo Fisher Scientific) and total cholesterol assay kit (Infinity^TM^ triglycerides kit, Thermo Fisher Scientific) respectively.

**Measurement of reactive oxygen species**

Fluorescence of reaction oxygen species (ROS) were detected by incubating frozen sections with 5 μM DCFDA for 30 min. Fluorescence intensity was first normalized to protein level and ROS activities were expressed as fold changes relative to Ctrl mice. MDA, an indicator of lipid peroxidation as a part of thiobarbituric acid reacting substances (TBARS), was assayed using TBARS Assay Kit (Caymen Chemicals) as instructed.

**Mitochondrial isolation and measurement of mitochondrial respiration**

Mitochondria were isolated from fresh ventricles and uncoupling and electron flow assays were performed using Seahorse XFe24 Analyzer as previously described^1^. Briefly, fresh ventricles were minced in ice-cold fiber relaxation buffer (KCl 100 mM, EGTA 5 mM, HEPES 5 mM, pH 7.0) and homogenized in ice-cold HES buffer (HEPES 5 mM, EDTA 1 mM, Sucrose 0.25 M, pH 7.4) in a glass douncer followed by differential centrifugation. Mitochondrial oxygen consumption rates (OCR) were assessed in respiration buffer (220 mM mannitol, 70 mM sucrose, 10 mM KH2PO4, 5 mM MgCl2, 2 mM HEPES, 1.0 mM EGTA, 1 mM EDTA, 0.2 % BSA, pH 7.2) containing 10 mM succinate (Complex II substrate) and 2 μM rotenone (Complex I inhibitor) with sequentially addition of 4 mM adenosine 5’-diphosphate (ADP) (Complex V substrate), 2.5 μg/ml oligomycin (Complex V inhibitor), 4 μM carbonyl cyanide 4-(trifluoromethoxy)phenylhydrazone (FCCP) (mitochondrial uncoupler for maximal respiration), and 4 μM antimycin A (Complex III inhibitor). Electron flow assays were performed by measuring basal OCR in the presence of 10 mM pyruvate, 2 mM malate and 4 μM FCCP, and after sequential addition of 2 μM rotenone, 10 mM succinate, 4 μM antimycin A, and 1 mM N,N,N’,N’-tetramethyl-p-phenylenediamine (TMPD)/10 mM ascorbate. Complex III respiration corresponds to the antimycin.A-sensitive respiration. OCR was normalized per microgram of mitochondrial protein.

**Mouse heart mitochondria DNA content**

Total DNA was extracted from ventricles and mitochondrial DNA (mtDNA) content was analyzed by RT-PCR of mtDNA-encoded 16s RNA (*MT-Rnr2*) to nuclear DNA (nDNA)-encoded hexokinase 2 (*Hk2*) intron 9 as described previously^2^.

**Isolation and culture of adult cardiomyocytes**

Adult mouse cardiomyocytes were isolated from 3-month-old male Ctrl and *Bscl2^cKO^* mice based on established procedures.^3^ The cardiomyocytes were suspended in plating media and plated onto laminin (5 µg/mL) precoated tissue culture plates. 1 h after plating, myocytes were changed to the culture media in the absence of 2,3-butanedione monoxime (BDM), ITS (Insulin/transferrin/selenium supplement) and lipid and kept in culture for 4 h, before exposure to isoproterenol (1 µM) for 20 mins.

**Tissue triglyceride and glycogen measurements**

For tissue TG enzymatic analyses, lipids were extracted from tissue homogenates and dissolved in chloroform. The concentrations of TG were measured using a triglyceride assay kit (Infinity^TM^ triglycerides kit, Thermo Fisher Scientific) and normalized to tissue weights as previously described.^11^

Ventricle glycogen content was measured by homogenizing tissues in 0.5 N KOH. Glycogen was then precipitated using ethanol and digested with amyloglycosidase (Sigma). The released glucose concentrations were then quantified using a glucose hexokinase assay kit (Thermo Fisher Scientific) and normalized to tissue weights as previously described.^4^

**Antibody information**

The following antibodies were used: rabbit antibodies against Phospho-PKA substrate (9624), HSL (4107), ATGL (2138), Phosphor-Phospholamban (Ser16/Thr17) (8496), Phospholamban (8495), HSP60 (12165) are from Cell Signaling Technology. CD36 (18836-1-AP), GAPDH (60004-1-IG), CPT1β (22170-1-AP), Prohibitin (10787-1-AP), SOD1 (10269-1-AP), SOD2 (24127-1-AP), Catalase (21260-1-AP), UCP2 (11081-1-AP) and UCP3 (10750-1-AP) are from Proteintech. Total OXPHOS Rodent WB Antibody Cocktail (ab110413) and PPARα (ab24509) are from AbCam.

**Supplemental Figures**

**
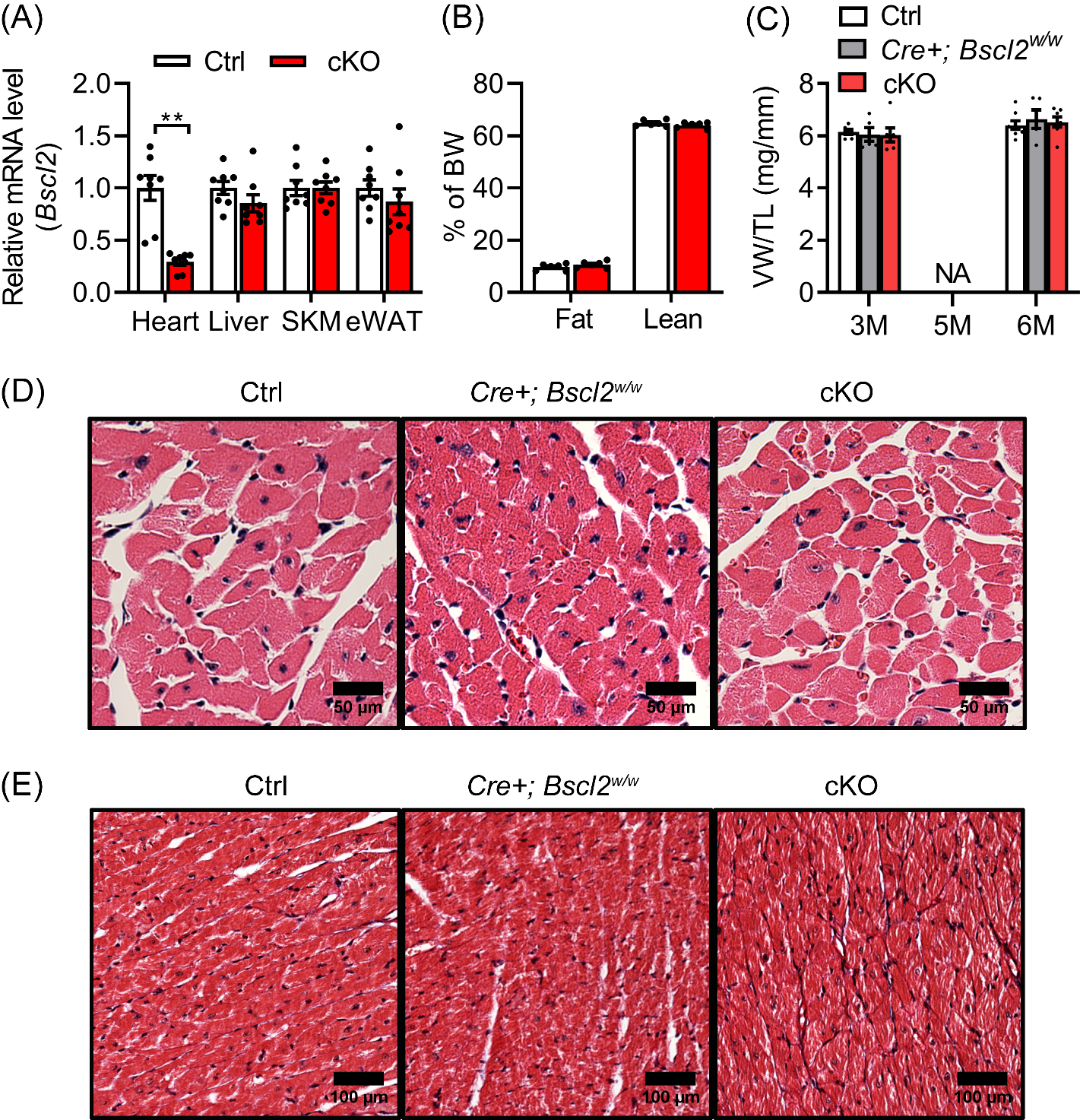
**

**Figure S1. Mice with cardiac-specific deletion of BSCL2 develop dilated cardiomyopathy.**

(A) RT-PCR analysis of *Bscl2* gene expression in heart, liver, skeletal muscle (SKM) and epididymal white adipose tissue (eWAT) of 3-month-old male *Cre-*; *Bscl2^f/f^* (Ctrl), *Cre+*; *Bscl2^w/w^*, and *Cre+*; *Bscl2^f/f^* (cKO) mice. *n* = 8 per group. **: *P* < 0.005 vs Ctrl, unpaired t tests. (B) % of fat and lean masses in 5-month-old male Ctrl and cKO mice. (C) Ventricle weight (VW) normalized to tibia length (TL) in 3-month-old (3M), 5-month-old and 6-month-old (6M) male Ctrl, *Cre+*; *Bscl2^w/w^* and cKO mice. 3M old: Ctrl, *n* = 8; *Cre+*; *Bscl2^w/w^*, *n* = 8; cKO, *n* = 12. 6M old: Ctrl, *n* = 11; *Cre+*; *Bscl2^w/w^*, *n* = 8; cKO, *n* = 9. (D) Representative images of hematoxylin-eosin staining of left ventricle apexes in male 6-month-old mice. Scale bar = 50 µm. (E) Histochemical assessment of fibrosis (trichrome staining) in male 6-months-old mice. Scale bar = 100 µm.


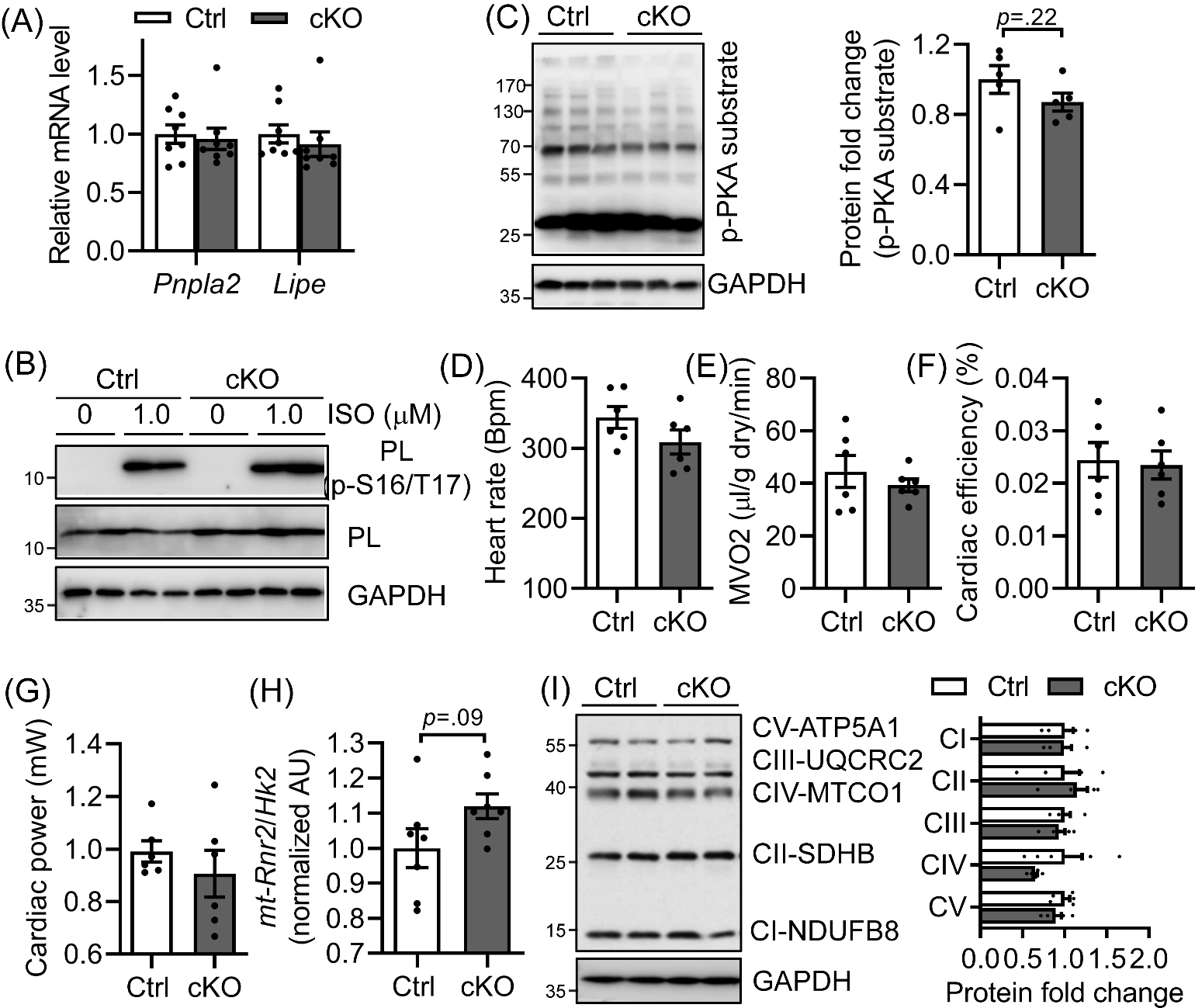


**Figure S2. Cardiac–specific deletion of BSCL2 regulates cardiac ATGL expression and substrate metabolism.**

(A) mRNA expression of lipolytic genes in 3-month-old *Cre-*; *Bscl2^f/f^* (Ctrl) and *Bscl2^cKO^* (cKO) mice. *n* = 8 per group. (B) Representative Western blotting to demonstrate phosphorylation of phospholamban (PL) 20 min after addition of 1 µM isoproterenol (ISO) in adult cardiomyocytes isolated from 3-month-old Ctrl and cKO mice. Two independent experiments. (C) Basal PKA-mediated substrate phosphorylation and quantification in homogenates from 3-month-old Ctrl and cKO ventricles. *n* = 3 per group. Three independent experiments. (D) Heart rate; (E) myocardial oxygen consumption (MVO2); (F) cardiac efficiency; and (G) cardiac power in *ex vivo* perfused working hearts. *n* = 6 per group. (H) Relative mtDNA content in ventricles assessed by RT-PCR and calculated from copy numbers of the mtDNA-encoded *mt-Rnr2* gene and the nuclear DNA-encoded *Hk2* intron 9 gene. Data were presented as fold change compared with Ctrl, arbitrarily defined as 1. *n* = 7 per group. (I) Representative Western blotting and quantification of mitochondrial complex proteins in ventricles of 3-month-old Ctrl and cKO mice. *n* = 5 per group. *: *P* < 0.05 with unpaired t test (parametric).


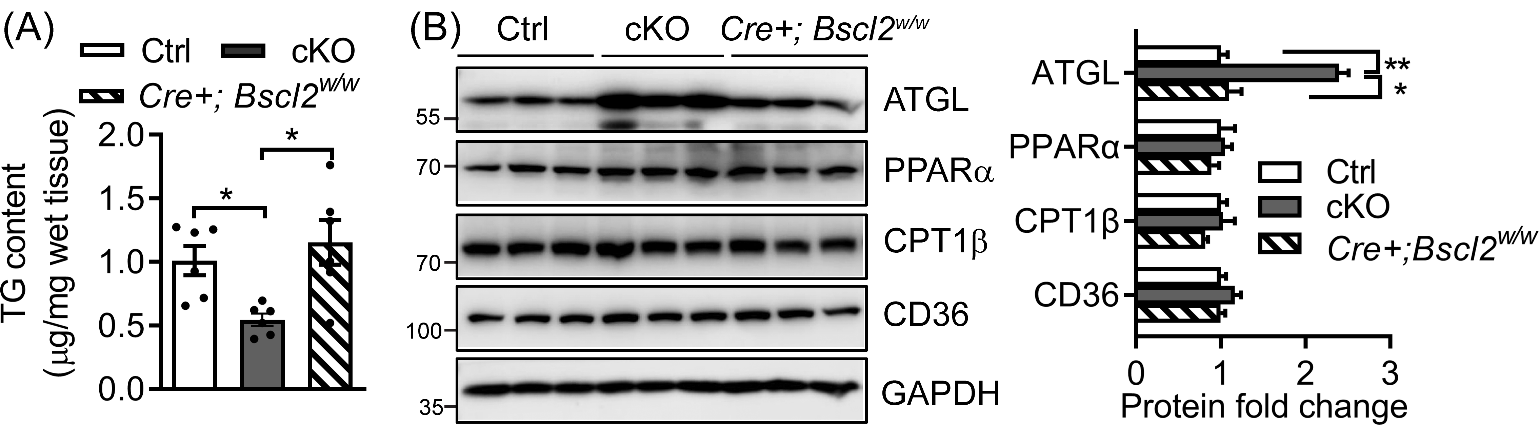


**Figure S3. 6 month-old *Bscl2^cKO^* mice exhibited cardiac lipid remodeling.**

(A) Enzymatic quantification of ventricular TG contents, *n* = 6 per group, One-way ANOVA followed by Tukey’s multiple comparisons test. (B) Representative Western blotting and quantifications in ventricles of 6-month-old *Cre-*; *Bscl2^f/f^* (Ctrl), *Bscl2^cKO^* (cKO) mice and *Cre+; Bscl2^w/w^* mice. *n* = 3 per group. Two independent experiments. Two-way ANOVA followed by Tukey’s multiple comparisons test. *: *P* < 0.05,


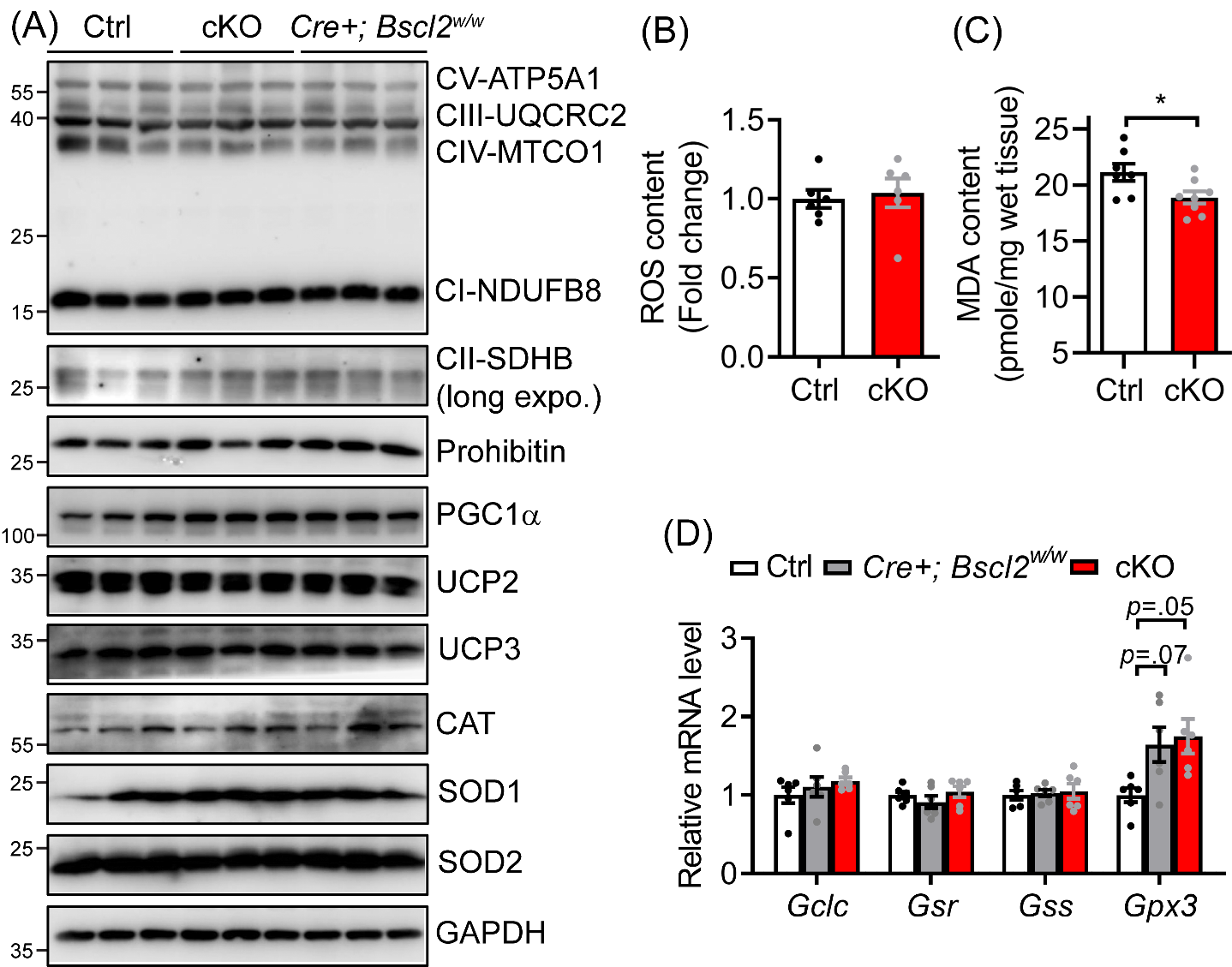


**Figure S4. Cardiac dysfunction in *Bscl2^cKO^* mice is not associated with mitochondrial dysfunction and oxidative stress.**

(A) Representative Western blotting in ventricles of 6-month-old *Cre-*; *Bscl2^f/f^* (Ctrl), *Bscl2^cKO^* (cKO) mice and *Cre+; Bscl2^w/w^* mice. *n* = 3 per group. Two independent experiments. (B) Ventricular ROS contents as measured by incubating ventricular homogenates with DCFDA dye. Data were presented as fold change with Ctrl normalized to 1. *n* = 6 per group. (C) Ventricular MDA contents as measured by TBARS kit. Ctrl, *n* = 7; cKO, *n* = 8. *: *P* < 0.05, unpaired t tests (parametric). (D) qRT-PCR analysis of glutathione metabolic genes in ventricles of 6-month-old Ctrl, cKO and *Cre+; Bscl2^w/w^* mice. *n* = 6 per group. Two-way ANOVA followed by Tukey’s multiple comparisons test.


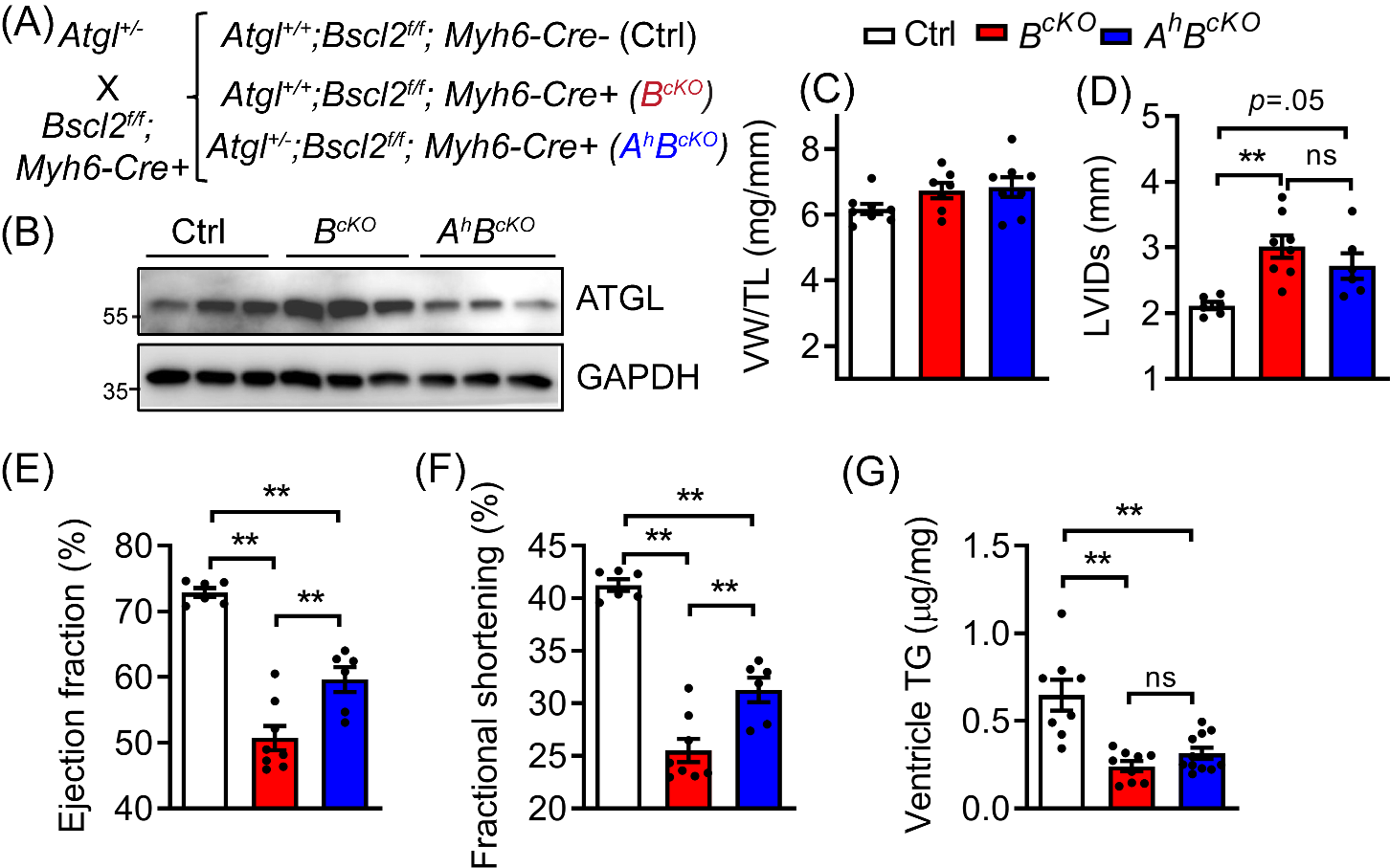


**Figure S5. ATGL haploinsufficiency partially rescues cardiac function of Bscl2^cKO^ mice.** Heterozygous *Atgl^+/-^* mice were bred with *Bscl2^cKO^* mice. Littermates as indicated in (A) were used for all experiments. (B) ATGL protein expression (*n* = 3), (C) ratios of ventricle weight (VW) to tibia length (TL), (D) left ventricle internal diameter at systole (LVIDs), (E) ejection fraction, (F) fractional shortening, (G) ventricle triglyceride (TG) contents normalized to tissue weights in male 6-month-old mice. Ctrl: *n* = 6, *B^cKO^*: *n* = 8; *A^h^B^cKO^*: *n* = 6. *: *P* < 0.05; **: *P* < 0.005. One-way ANOVA with Tukey’s multiple comparisons test. ns: not significant.


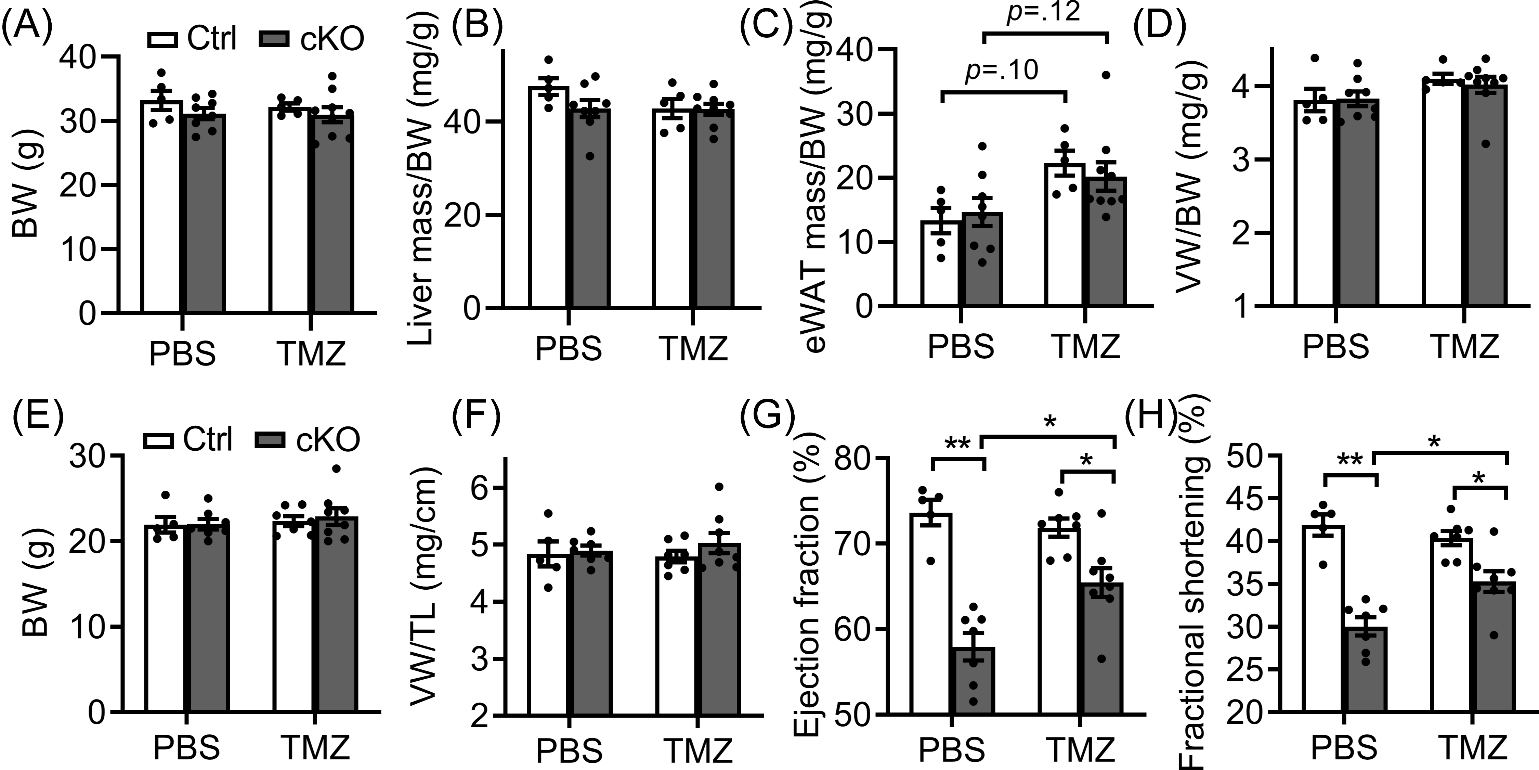


**Figure S6. Inhibition of fatty acid oxidation partially rescues cardiac function in *Bscl2^cKO^* mice.**

6-month-old male *Cre-*; *Bscl2^f/f^* (Ctrl) and *Bscl2^cKO^* (cKO) mice were daily i.p. injected with PBS or trimetazidine (TMZ) at 15 mg/kg body weights (BW) for a total of 6 weeks. (A) Body weight (BW); (B) ratio of liver mass to BW; (C) ratio of epididymal white fat (eWAT) to BW and (D) ratio of ventricle weight (VW) to BW at the end of treatment. For A-D, PBS-Ctrl, *n* = 5; PBS-cKO, *n* = 8. TMZ-Ctrl, *n* = 5, TMZ-cKO, *n* = 9. (E-H) 6 month-old female Ctrl and cKO mice were treated with TMZ as above. (E) BW; (F) ratio of ventricle weight (VW) to tibia length (TL); (G) ejection fraction and (H) fractional shortening were assessed after 6 weeks of PBS or TMZ injection. For E-H, PBS-Ctrl, *n* = 5; PBS-cKO, *n* = 7. TMZ-Ctrl, *n* = 7, TMZ-cKO, *n* = 8. *: *P* < 0.05; **: *P* < 0.005. Two-way ANOVA followed by Tukey’s post-hoc tests.

**Supplemental Tables**

**Table S1. Quantitative real-time PCR primer sequences for murine genes**

| **Gene name** |  | **Primer sequence** | **Gene name** |  | **Primer sequence** |
| --- | --- | --- | --- | --- | --- |
| ***36B4*** | 5F | CGCTTTCTGGAGGGTGTCCGC | ***Gsr*** | 5F | CAGATGTTGACTGCCTGCTC |
|  | 3R | TGCCAGGACGCGCTTGTACC |  | 3R | GACGTCTCCCACAGCATAGAC |
| ***Acadl*** | 5F | AGCCTCCGTGGAGTTGCACA | ***Gss*** | 5F | CTTGCTGCTCCTAGCCACTT |
|  | 3R | CCAGGAACTACGTGAAGCAAAG |  | 3R | AGGCTCTCTCCTCACTGTCCT |
| ***Actb*** | 5F | GACGGCCAGGTCATCACTAT | ***Lipe*** | 5F | GCTCTTCTTCGAGGGTGATG |
|  | 3R | CTTCTGCATCCTGTCAGCAA |  | 3R | ACACTGAGGCCTGTCTCGTT |
| ***Acox1*** | 5F | CCTGACAGAAGCCTACAAG | ***Lpl*** | 5F | CCTAAGGACCCCTGAAGAC |
|  | 3R | TGTCTTGAATCTTGGGGAGTT |  | 3R | GACATTGGAGTCAGGTTCTC |
| ***Bscl2*** | 5F | GCTCTTCTGCACCATCCTTC | ***Myh6*** | 5F | CGCCTATGAGGAGTCTCTGG |
|  | 3R | CGGTGGAGGAATCACAGTC |  | 3R | TTCTCCACCTCCAGCTGTTT |
| ***Cd36*** | 5F | CGTTTCAACTCTCACACACATAAG | ***Myh7*** | 5F | CGCCTATGAGGAGTCTCTGG |
|  | 3R | TGAGACTCTGAAAGGATCAGCA |  | 3R | TCCAGTTGCTTTCGGATCTT |
| ***Cpt1β*** | 5F | TTGCCCTACAGCTGGCTCATTTCC | ***Nppa*** | 5F | ATTGACAGGATTGGAGCCCAGAGT |
|  | 3R | GCACCCAGATGATTGGGATACTGT |  | 3R | TGACACACCACAAGGGCTTAGGAT |
| ***Col1a2*** | 5F | GTCCTAGTCGATGGCTGCTC | ***Nppb*** | 5F | ATCTCCTGAAGGTGCTGTCC |
|  | 3R | GTCAGCACCACCAATGTCC |  | 3R | AGCTGTCTCTGGGCCATTT |
| ***Dgat1*** | 5F | TGGCCAGGACAGGAGTATTT | ***Pdk4*** | 5F | CTCCTTCGGTGCAGCTGG |
|  | 3R | CACAGCTGCATTGCCATAGT |  | 3R | GTCCACTGTGCAGGTGTCT |
| ***Dgat2*** | 5F | CTTCCTGGTGCTAGGAGTGG | ***Pgc1α*** | 5F | CCCTGCCATTGTTAAGACC |
|  | 3R | CCAGCTGGATGGGAAAGTAG |  | 3R | TGCTGCTGTTCCTGTTTTC |
| ***Gclc*** | 5F | GGATTCACACTGCCAGAACA | ***Pnpla2*** | 5F | GATGTGCAAACAGGGCTACA |
|  | 3R | TGGCACATTGATGACAACCT |  | 3R | CTTCCTCTGCATCCTCTTCC |
| ***Gdf15*** | 5F | AGCCGAGAGGACTCGAAC | ***Pparα*** | 5F | CCACGAAGCCTACCTGAAGA |
|  | 3R | GTTGACGCGGAGTAGCAG |  | 3R | ACTGGCAGCAGTGGAAGAAT |
| ***Glut1*** | 5F | GCTTTGTGGCCTTCTTTGAA | ***Ppia*** | 5F | CTGTTTGCAGACAAAGTTCCA |
|  | 3R | AAGAAGAGCACGAGGAGCAC |  | 3R | AGGATGAAGTTCTCATCCTCA |
| ***Glut4*** | 5F | CGGCTCTGACGATGGGGA | ***Tfam*** | 5F | CCACAGAACAGCTACCCAAA |
|  | 3R | GGTGCCTTGTGGGATGGA |  | 3R | CATCAGCTGACTTGGAGTTA |
| ***Gpx3*** | 5F | CCATTTGGCTTGGTCATTCT |  |  |  |
|  | 3R | CCCGTTCACATCTCCTTTCT |  |  |  |

**Table S2. Echocardiography in mice under normal chow diet.**

| **Age** | **3-month-old** | | | **5-month-old** | | | **6-month-old** | | |
| --- | --- | --- | --- | --- | --- | --- | --- | --- | --- |
| **Genotype** | **Ctrl**  **(*n* = 8)** | ***Cre+; Bscl2^w/w^* (*n* = 6)** | **cKO**  **(*n* = 12)** | **Ctrl**  **(*n* = 6)** | ***Cre+; Bscl2^w/w^* (*n* = 8)** | **cKO**  **(*n* = 10)** | **Ctrl**  **(*n* = 11)** | ***Cre+; Bscl2^w/w^* (*n* = 8)** | **cKO**  **(*n* = 9)** |
| **BW (g)** | 26.6±0.8 | 26.9±0.4 | 26.8±0.5 | 27.7±0.4 | 28.5±1 | 28.7±0.8 | 29.5±0.6 | 32.1±1.74 | 30.6±0.6 |
| **Heart rate** | 572±14 | 622±9 | 564±18 | 628±8 | 599±11 | 594±9 | 622±10 | 585±5 | 588±11 |
| **LVAWd (mm)** | 0.71±0.03 | 0.8±0.03 | 0.76±0.03 | 0.96±0.01 | 0.88±0.03 | 0.83±0.03* | 0.82±0.02 | 0.75±0.04 | 0.69±0.06* |
| 3, 5 and 6-month old male *Cre-; Bscl2^f/f^* (Ctrl), *Cre+; Bscl2^w/w^* and *Cre+; Bscl2^f/f^* (*Bscl2^cKO^*, simplified as cKO) mice were kept under normal chow diet. BW: body weights; LVAWd: left ventricle anterior wall thickness at end diastole. *: *P* < 0.05 vs Ctrl mice within the same age group. Two-way ANOVA with Tukey’s multiple comparisons test. | | | | | | | | | |

**Table S3. Plasma parameters and echocardiography in mice fed with normal chow and high fat diets.**

| **Diet** | **NCD** | | **HFD** | |
| --- | --- | --- | --- | --- |
| **Genotype** | **Ctrl** | **cKO** | **Ctrl** | **cKO** |
| **Body weight (g)** | 32.1±0.6 | 31.4±0.5 | 43.7±1.2^##^ | 45.7±0.7^##^ |
| **Glucose (mg/dL)** | 153±5 | 152±4 | 233±8^##^ | 221±10^##^ |
| **TG (mg/dL)** | 59.2±4.9 | 74.1±3.9 | 53.2±1.2 | 61.9±1.7 |
| **TC (mg/dL)** | 82±9.2 | 64.4±6.3 | 207.5±10.8^##^ | 203.7±7.8^##^ |
| **NEFA (mM)** | 0.49±0.07 | 0.35±0.02 | 1.38±0.12^##^ | 1.42±0.1^##^ |
| **Glycerol (mg/dL)** | 19.8±1.9 | 25.3±2.9 | 47.1±4^#^ | 43.8±3.4^#^ |
| **Heart rate** | 600±12 | 588±10 | 610±7 | 590±8 |
| **LVPWd (mm)** | 0.83±0.06 | 0.76±0.04 | 0.92±0.06 | 0.80±0.03 |
| **LVPWs (mm)** | 1.39±0.06 | 1.15±0.05* | 1.55±0.03 | 1.42±0.05^##^ |
| **LVAWd (mm)** | 0.77±0.04 | 0.68±0.05 | 0.95±0.03^#^ | 0.83±0.02^#^ |
| **LVAWs (mm)** | 1.4±0.04 | 1.15±0.05** | 1.55±0.05 | 1.39±0.03*^##^ |
| **LVIDd (mm)** | 3.84±0.07 | 4.11±0.12 | 3.84±0.16 | 4.14±0.08 |
| **EF (%)** | 72.6±0.75 | 56.8±1.13** | 70.4±1.42 | 67.9±1.0**^##^ |
| Body weights, plasma biochemistry and echocardiography were analyzed in ad-libitum 6 month-old male *Cre-; Bscl2^f/f^* (Ctrl) and *Bscl2^cKO^* (cKO) mice fed with normal chow diet (NCD) or high fat diet (HFD) starting at 3 months old for additional 3 months. TG: triglyceride; TC: total cholesterol; NEFA: nonesterified fatty acid. TG: triglyceride; TC: total cholesterol; NEFA: nonesterified fatty acid. LVPWd: left ventricle post wall thickness at end diastole; LVPWs: Left ventricle post wall thickness at end systole; LVAWd: left ventricle anterior wall thickness at end diastole; LVAWs: left ventricle anterior wall thickness at end systole; LVIDd: left ventricle internal diameter at end diastole; EF: ejection fraction. Data were presented as means ± SEM. For plasma parameters, NCD-Ctrl, *n* = 9; NCD-cKO, *n* = 9; HFD-Ctrl, *n* = 7; HFD-cKO, *n* = 11. For echocardiography, NCD-Ctrl, *n* = 9; NCD-cKO, *n* = 12; HFD-Ctrl, *n* = 12; HFD-cKO, *n* = 16. *: *P*<0.05; **: *P* < 0.005 vs Ctrl mice under the same diet. #: *P* < 0.05; ##: *P* < 0.005 vs NCD-fed same genotype. Two-way ANOVA with Tukey’s multiple comparisons tests. | | | | |

**Table S4. Echocardiography in mice with partial deletion of ATGL and cardiac-specific deletion of *Bscl2*.**

| **Genotype** | **Ctrl (*n* = 6)** | ***B^cKO^* (*n* = 8)** | ***A^h^B^cKO^* (*n* = 6)** |
| --- | --- | --- | --- |
| **BW (g)** | 29.1±0.5 | 30.6±0.7 | 30.6±0.9 |
| **Heart rate** | 619±13 | 592±11 | 611±6 |
| **LVPWd (mm)** | 0.82±0.03 | 0.71±0.05 | 0.74±0.04 |
| **LVPWs (mm)** | 1.32±0.04 | 1.07±0.08* | 1.17±0.04 |
| **LVAWd (mm)** | 0.79±0.03 | 0.66±0.05* | 0.67±0.04 |
| **LVAWs (mm)** | 1.39±0.06 | 1.04±0.06** | 1.14±0.04* |
| **LVIDd (mm)** | 3.67±0.10 | 4.03±0.18* | 3.94±0.15 |
| 6-month-old male *Atgl^+/+^*;*Bscl2^f/f^*;*Cre-* (Ctrl), *Atgl^+/+^;Bscl2^f/f^*;*Cre+* (*B^cKO^*), and *Atgl^+/-^*;*Bscl2^f/f^*;*Cre+* (*A^h^B^cKO^*) mice were kept under normal chow diet. BW: body weights; LVPWd: left ventricle post wall thickness at end diastole; LVPWs: Left ventricle post wall thickness at end systole; LVAWd: left ventricle anterior wall thickness at end diastole; LVAWs: left ventricle anterior wall thickness at end systole; LVIDd: left ventricle internal diameter at end diastole. *: *P* < 0.05; **: *P* < 0.005 vs Ctrl mice within the same age group. One-way ANOVA with Tukey’s multiple comparisons test. | | | |

**Table S5. Plasma parameters and echocardiography in mice treated with trimetazidine.**

| **Treatment** | **PBS** | | **TMZ** | |
| --- | --- | --- | --- | --- |
| **Genotype** | **Ctrl (*n* = 10)** | **cKO (*n* = 9)** | **Ctrl (*n* = 6)** | **cKO (*n* = 9)** |
| **Glucose (mg/dL)** | 116±3.4 | 116.3±5.2 | 122.6±4.3 | 128.2±5.2 |
| **TG (mg/dL)** | 81±7.5 | 78.5±5.6 | 75.7±5 | 69.5±3.6 |
| **TC (mg/dL)** | 71±7.1 | 61.3±7.1 | 90.2±6.2^#^ | 78.6±3.5^#^ |
| **NEFA (mM)** | 0.76±0.24 | 0.66±0.12 | 0.6±0.13 | 0.49±0.08 |
| **Glycerol (mg/dL)** | 13.7±0.9 | 14.3±0.9 | 14.4±0.8 | 15±0.9 |
| **Heart rate** | 641±8 | 623±7 | 619±10 | 616±7 |
| **LVPWd (mm)** | 0.72±0.05 | 0.66±0.03 | 0.81±0.05 | 0.63±0.03** |
| **LVPWs (mm)** | 1.24±0.10 | 1.03±0.06* | 1.34±0.07 | 1.20±0.04^#^ |
| **LVAWd (mm)** | 0.78±0.03 | 0.71±0.05 | 0.80±0.03 | 0.78±0.04^#^ |
| **LVAWs (mm)** | 1.46±0.03 | 1.11±0.06** | 1.44±0.03 | 1.27±0.04 |
| **LVIDd (mm)** | 3.84±0.06 | 4.12±0.10* | 3.82±0.15 | 4.11±0.05 |
| Plasma biochemistry and echocardiography were analyzed in ad-libitum 7.5 month-old male *Cre-; Bscl2^f/f^* (Ctrl) and *Bscl2^cKO^* (cKO) mice i.p. injected with PBS or trimetazidine (TMZ) for 6 weeks starting at 6 months old. TG: triglyceride; TC: total cholesterol; NEFA: nonesterified fatty acid. LVPWd: left ventricle post wall thickness at end diastole; LVPWs: left ventricle post wall thickness at end systole; LVAWd: left ventricle anterior wall thickness at end diastole; LVAWs: left ventricle anterior wall thickness at end systole; LVIDd: left ventricle internal diameter at end diastole. Data were presented as means ± SEM. *: *P* < 0.05; **: *P* < 0.005 vs Ctrl mice under the same treatment. #: *P* < 0.05; ##: *P* < 0.005 vs PBS treated same genotype. Two-way ANOVA with Tukey’s multiple comparisons tests. | | | | |

**References**

1. Zhou H, Lei X, Yan Y*, et al.* Targeting ATGL to rescue BSCL2 lipodystrophy and its associated cardiomyopathy. *JCI Insight* 2019;**4**:e129781.

2. Zhou H, Black SM, Benson TW, Weintraub NL, Chen W. Berardinelli-Seip Congenital Lipodystrophy 2/Seipin Is Not Required for Brown Adipogenesis but Regulates Brown Adipose Tissue Development and Function. *Mol Cell Biol* 2016;**36**:2027-2038.

3. Ackers-Johnson M, Li PY, Holmes AP*, et al.* A Simplified, Langendorff-Free Method for Concomitant Isolation of Viable Cardiac Myocytes and Nonmyocytes From the Adult Mouse Heart. *Circ Res* 2016;**119**:909-920.

4. Xu W, Zhou H, Xuan H*, et al.* Novel metabolic disorders in skeletal muscle of Lipodystrophic Bscl2/Seipin deficient mice. *Mol Cell Endocrinol* 2019;**482**:1-10.
